# Iodinated Near-Infrared Dyes as Effective Photosensitizers for the Photodynamic Eradication of Amphotericin B-Resistant Candida Pathogens

**DOI:** 10.3390/molecules30234652

**Published:** 2025-12-04

**Authors:** Chen Damti, Andrii Bazylevich, Amartya Sanyal, Olga Semenova, Arjun Prakash, Iryna Hovor, Bat Chen R. Lubin, Leonid Patsenker, Gary Gellerman

**Affiliations:** 1 Department of Chemical Sciences, Faculty of Natural Sciences, Ariel University, Ariel 40700, Israel; chendrori27@gmail.com (C.D.); andriib@ariel.ac.il (A.B.); amartya@ariel.ac.il (A.S.); olgasem@ariel.ac.il (O.S.); arjunvprakash22@gmail.com (A.P.); irynah@ariel.ac.il (I.H.); 2 Department of Chemical Engineering, Biotechnology and Materials, Ariel University, Ariel 40700, Israel; revital5@gmail.com; 3 Agriculture and Ecology Department, Eastern Regional R&D Center, Ariel 40700, Israel; 4 Independent Researcher, Ariel 40700, Israel; leonid.patsenker@gmail.com

**Keywords:** antifungal photodynamic therapy, iodinated cyanines, iodinated methylene blue, *Candida albicans*, *Candida parapsilosis*, amphotericin B

## Abstract

**Amphotericin:** B (**AmpB**)-resistant *Candida* (*C*.) species, such as *C*. *parapsilosis*, are among the most common causes of invasive fungal infections, posing significant challenges in hospital settings. Although **AmpB** is considered the first-line treatment owing to its broad-spectrum fungicidal activity, its use is hampered by severe side effects and the emergence of acquired resistance, particularly in *C*. *parapsilosis*, which exhibits reduced susceptibility to polyene, azole, and echinocandin-based antifungal drugs. Here, we present findings on photodynamic therapy (PDT) that targets the opportunistic fungal pathogens *C*. *parapsilosis* and *C*. *albicans* via the use of photosensitizers from the iodocyanine and newly developed iodinated Methylene blue families. These compounds contain heavy iodine atoms that increase the production of reactive oxygen species (ROS), the agents responsible for oxidative cellular damage, via the heavy-atom effect, which promotes intersystem crossing (ISC) and triplet-state formation. A strong antifungal effect was observed against **AmpB**-resistant *C*. *parapsilosis*, indicating a correlation between the quantum yield of ROS generation and the photosensitizing efficacy under near-infrared (NIR) light irradiation. The combination of efficient cellular uptake and enhanced ROS generation positions iodinated photosensitizers as promising candidates for the treatment of drug-resistant *Candida* strains.

## 1. Introduction

Fungal infections have emerged as a significant public health concern, particularly in immunocompromised individuals. Opportunistic fungal pathogens such as *Candida*, *Aspergillus*, and *Cryptococcus* are responsible for a substantial number of infections worldwide. *Candida* species are among the most prevalent causes of invasive fungal infections, with *Candida parapsilosis* (*C*. *parapsilosis*) and *Candida albicans* (*C*. *albicans*) being particularly problematic in hospital settings [1]. Epidemiological data indicate that invasive candidiasis accounts for approximately 1.57 million cases annually, with mortality rates reaching 63.6% [2]. Furthermore, it has been estimated that invasive fungal infections cause more than 3.8 million deaths annually, with a significant proportion of these deaths resulting from undiagnosed and untreated cases [2]. The growing prevalence of fungal infections is attributed to several factors, including climate change, increased use of immunosuppressive therapies, and an increasing population of immunocompromised individuals [3]. Additionally, changes in the epidemiology, diversity in clinical presentation, and emergence of new pathogens further complicate the management and understanding of fungal diseases [4]. These infections can affect various organ systems, leading to conditions such as bloodstream infections (candidemia), respiratory infections (aspergillosis), and central nervous system infections (cryptococcal meningitis). Recent studies suggest that fungal diseases cause more deaths annually than malaria or breast cancer, highlighting the urgent need for improved diagnostics and treatment strategies [5].

The genus *Candida* belongs to the Ascomycota phylum, within the Saccharomycotina subphylum, and is classified under the Saccharomycetales order [6]. Phylogenetic studies have shown that *Candida* species are polyphyletic and have evolved independently to occupy similar ecological niches as opportunistic pathogens. This evolutionary adaptability contributes not only to their ability to colonize diverse host environments, but also to the development of drug resistance traits [7]. To effectively cause mucosal and/or systemic disease and withstand the subsequent antifungal host response and antifungal drug treatment, *Candida* employs several virulence factors, including temperature adaptation, adhesion and invasion, nutrient acquisition, immune evasion, and drug tolerance [8].

Infections caused by *Candida* species are typically treated with four main classes of antifungal drugs: polyenes, azoles, echinocandins, and pyrimidine analogs. One of the most commonly used drugs is Amphotericin B (**AmpB**), a member of the polyene class, which is considered a first-line treatment for severe fungal infections because of its broad-spectrum fungicidal activity. The choice of treatment depends on factors such as species-specific susceptibility and patient health status. Despite the availability of these antifungal agents, increasing resistance to these treatments has been documented, particularly in *C*. *parapsilosis*, which has shown a marked decrease in susceptibility to polyenes, azoles, and echinocandin-based drugs [5,9,10].

Antifungal resistance has become a major challenge in clinical treatment, limiting the effectiveness of currently available drugs. Resistance develops when fungal pathogens adapt and survive exposure to antifungal agents, reducing drug efficacy and complicating treatment. Several mechanisms contribute to antifungal resistance, including genetic mutations that alter drug targets, increased expression of efflux pumps that remove antifungal agents from fungal cells, and the formation of biofilms that act as protective barriers against treatment [5,11,12].

The overuse of antifungal drugs, particularly in preventive treatments, has accelerated the development of resistance by selectively promoting the survival of resistant strains [11]. A particularly concerning development is the rise of multidrug-resistant (MDR) fungal species, such as *C. auris*, which exhibit resistance to multiple drug classes, including azoles, echinocandins, and polyenes [5]. These resistant pathogens are more difficult to eliminate, leading to higher mortality rates and prolonged infections in patients.

**AmpB** is a polyene antifungal agent that has been widely used for the treatment of invasive fungal infections. It binds to ergosterol, a major component of fungal cell membranes, leading to increased membrane permeability and cell death [13]. **AmpB** is especially valuable in treating infections caused by fungi that are resistant to other antifungal drugs [14]. Despite its effectiveness, **AmpB** is associated with several adverse effects that limit its clinical use. The most notable among these factors is nephrotoxicity, which can result in significant kidney impairment. Other side effects include infusion-related reactions such as fever and chills, as well as disturbances in serum electrolytes [15].

Photodynamic therapy (PDT) is a non-invasive approach that has garnered increasing interest in circumventing conventional drug resistance and is a viable option for addressing pathogen infections, especially those resistant to traditional medicines. PDT employs a photosensitizer (PS), which, upon activation by light, generates highly toxic, oxygen-based (ROS) and non-oxygen-based reactive species (RSs), such as singlet oxygen, superoxide, hydrogen peroxide, and free radicals [16]. These RSs induce oxidative damage critical to cellular components, leading to microbial cell death [16,17]. Importantly, the non-specific oxidative mechanism of PDT minimizes the risk of resistance development, making it an attractive modality for treating persistent and resistant infections [18].

To date, a number of PSs of different dye classes have been introduced and evaluated. The majority of these dyes, such as cyanines (e.g., Indocyanine green, ICG), phenothiazinium dyes (e.g., Methylene blue and Toluidine blue), and BODIPY derivatives, are cationic fluorophores.

Iodinated heptamethine cyanine dyes are relatively new but well-characterized photosensitizers. The incorporation of iodine atoms in conventional (non-iodinated) cyanines enhances intersystem crossing via the heavy-atom effect, thereby increasing the efficiency of ROS generation upon photoactivation. These dyes exhibit, therefore, high quantum yields of singlet oxygen and the generation of other reactive species [19,20,21] but are also characterized by strong absorption in the near-infrared (NIR) optical window, which is advantageous for in vivo applications, as light penetration through the body is maximized and light absorption and scattering are minimized, causing less damage to the skin [22] and improving safety for therapeutic use [23]. Our recent studies highlighted the phototoxic potential of iodinated cyanines against bacterial pathogens [21,24,25,26,27,28] and cancer cells [29]. However, the antifungal activity of these dyes, particularly against highly drug-resistant *Candida* strains remains unexplored.

Methylene blue (**MB**), categorized as a phenothiazine dye, is another example of a well-known and thoroughly investigated PS with less pronounced but still satisfactory absorption in the deep-red region and high singlet oxygen production [30,31]. The high efficacy of **MB** in eradicating pathogenic bacteria [32], fungi such as *C*. *albicans* [33,34,35,36], *C*. *parapsilosis* [35], *C*. *glabrata* [35], and cancer cells [37,38] has been repeatedly reported in the literature. We anticipated that, similar to cyanines and some other dye classes, the introduction of iodine atoms in **MB** can improve its ROS generation and photocytotoxicity. However, such iodinated Methylene blue derivatives have never been synthesized and investigated.

In the present study, for the first time, we developed a simple one-step synthetic approach and synthesized two iodinated **MB** derivatives—monoiodo (**IMB**) and diiodo-(**2IMB**) Methylene blue dyes. The photodynamic efficacy of these dyes and iodinated heptamethine cyanines was investigated against clinically relevant *C*. *albicans* and antifungal drug-resistant *C*. *parapsilosis*.

## 2. Results

### 2.1. Synthesis of Photosensitizers

**Scheme 1:**
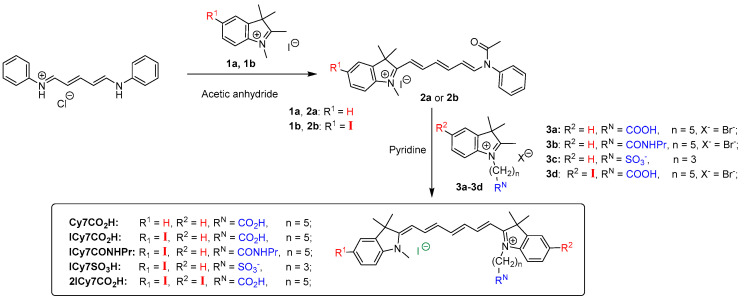
Synthesis of heptamethine cyanine dyes.

The heptamethine cyanine dyes **Cy7CO_2_H**, **ICy7CO_2_H**, **ICy7CONHPr**, **ICy7SO_3_H**, and **2ICy7CO_2_H** were synthesized according to previously reported procedures [21,28] with reasonable yields (17–56%) via a one-pot sequential reaction of *N*-[5-(phenylamino)-2,4-pentadienylidene]aniline with the first indolenine molecule **1a** or **1b** in acetic anhydride to form a corresponding *N*-phenylacetamide derivative **2a** or **2b**, which was then reacted with the second indolenine **3a**–**3d** in the presence of pyridine (Scheme 1).

**Scheme 2:**
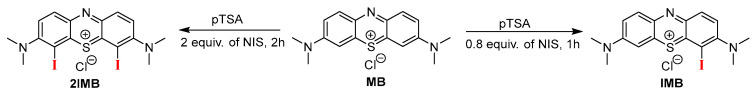
Synthesis of monoiodo-(**IMB**) and diiodo-(**2IMB**) substituted Methylene blue dyes.

The iodinated **MB** dyes **IMB** and **2IMB** were synthesized in reasonable yields (67% for **IMB** and 43% for **2IMB**) via direct iodination of **MB** with *N*-iodosuccinimide (NIS) in the presence of *p*-toluene sulfonic acid (pTSA) at room temperature (Scheme 2). The preferable yield of the mono- or di-iodinated products was achieved by varying the ratio between **MB** and the iodination agent.

### 2.2. Spectral Properties

**Figure 1:**
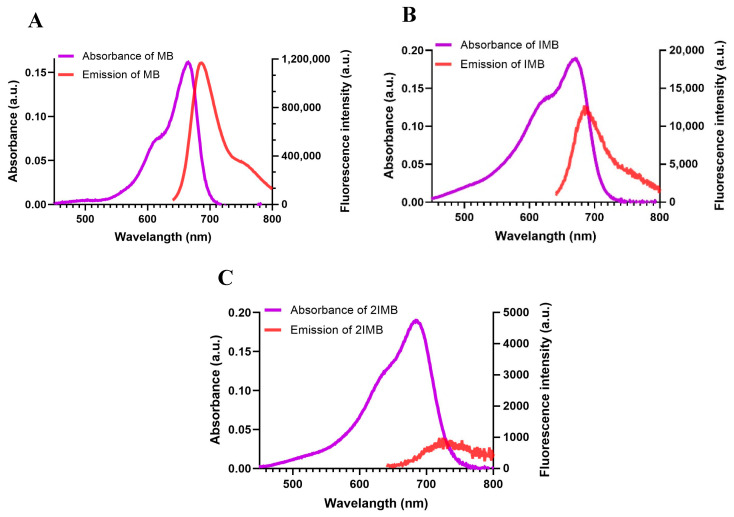
Absorption and fluorescence spectra of (**A**) **MB**, (**B**) **IMB** and (**C**) **2IMB** in saline.

The absorption and emission spectra of the newly synthesized **MB** derivatives are presented in Figure 1. The absorption (λ_max_Ab) and fluorescence (λ_max_Fl) maxima, extinction coefficients (ε), absolute fluorescence quantum yields (Φ_F_), relative quantum yields of ROS generation (φ_ROS_) (determined using the H_2_DCFDA-based method), and absolute quantum yields of singlet oxygen generation (Φ_Δ_), measured via DPBF for the investigated cyanine and **MB** derivatives (*c*_Dye_~1 μM), are summarized in Table 1 and Table 2, respectively. To avoid confusion between relative and absolute quantum yields, Φ_F_ and Φ_Δ_ (absolute values) are given as percentages, whereas φ_ROS_ (relative values) are expressed as a fraction of one.

The cyanine dyes demonstrate longer-wavelength absorption in the fNIR range (740–754 nm) and significantly higher extinction coefficients (109,000–238,000 M^−1^cm^−1^) compared to **MB** derivatives (λ_max_Ab = 665–685 nm, ε = 24,000–74,200 M^−1^cm^−1^) in saline and somewhat higher extinction coefficients in MeOH (ε = 30,000–88,200 M^−1^cm^−1^), making them easily excitable within the biological window. Additionally, cyanines emit at longer wavelengths and have increased fluorescence quantum yields in aqueous media (Φ_F_~4–13%) compared with **MB** (Φ_F_ = 2%). The brightness of cyanines (*B*~6800–23,800 M^−1^cm^−1^), calculated as *B* = ε × Φ_F_, is nearly 4.5–16 times higher than that of **MB** (*B*~1500 M^−1^cm^−1^), whereas iodinated **IMB** and **2IMB** derivatives exhibit negligible practical fluorescence. Importantly, iodination has only a minor effect on the ε, Φ_F_, and *B* values of cyanines. These properties facilitate the application of iodinated cyanines for fluorescence imaging of these PSs in vivo, including tracking their delivery and accumulation in target tissues.

Conversely, iodinated **MB** derivatives exhibit extremely low fluorescence in aqueous saline, with Φ_F_ values of ~0.021% (**IMB**) and 0.0015% (**2IMB**), along with reduced extinction coefficients, rendering fluorescence monitoring impractical. The decrease in Φ_F_ and ε upon iodination of **MB** likely results from enhanced intersystem crossing (ISC) to the triplet state and aggregation of these dyes in aqueous saline, as evidenced by an increased shoulder on the shorter-wavelength slope of the absorption spectrum (Figure 1). Nevertheless, these dyes exhibit significant singlet oxygen generation yields (Φ_Δ_~20–52% in acetonitrile), comparable in magnitude to those of cyanines (Φ_Δ_~4.5–65%) (see Section 2.4).

### 2.3. Photostability of Cyanines and Methylene Blue (MB) Derivatives

**Figure 2:**
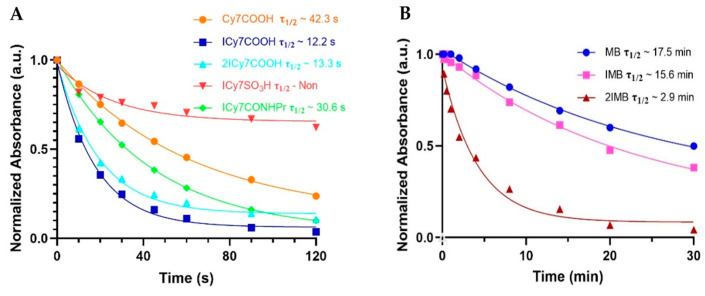
Decay profiles (normalized absorbances vs. illumination time) for cyanines (**A**) and **MB** derivatives (**B**) in saline, and the corresponding half-lives (τ_1/2_). Irradiation with 730 nm and 632 nm LEDs, respectively; light power density of 75 mW/cm^2^. The half-life (τ_1/2_) value of **ICy7SO_3_H** could not be determined within the experimental time range.

The photostabilities of cyanines and **MB**-based dyes were evaluated in saline under continuous light irradiation, using 730 nm and 632 nm LEDs for cyanines and **MB** derivatives, respectively. The light power density was 75 mW/cm^2^, identical to that used in the subsequent biological experiments. Absorption spectra were recorded, and the normalized absorbances at λ_max_Ab were plotted as a function of illumination time, fitted with a monoexponential function (Figure 2A,B), and used to estimate photostabilities through the decay half-lives (τ_1/2_).

The cyanine dyes exhibited distinct differences in photostability, increasing in the order: **2ICy7COOH** ≈ **ICy7COOH** < **ICy7SO_3_H** < **ICy7CONHPr** < **Cy7COOH**. Thus, the most photostable dye is **Cy7COOH**, while iodination facilitates photobleaching, likely due to the increasing population of the long-lived triplet state and increased probability of photochemical reactions. The higher stability of **ICy7CONHPr** compared with iodinated cyanines bearing negatively charged carboxylic groups suggests that the neutral amide group moderates the oxidative degradation rate by influencing dye–solvent interactions and restricting the access of reactive oxygen species to the polymethine chain. In contrast, the sulfonated derivative **ICy7SO_3_H**, which is highly polar and anionic, is more exposed to the aqueous environment, facilitating ROS-mediated oxidation. The overall trend demonstrates the intrinsic trade-off between photodynamic efficiency and photochemical stability: substituents promoting intersystem crossing and ROS generation simultaneously increase the vulnerability of the chromophore to photodecomposition.

The photostabilities decreased upon iodination in the following order: **MB** (τ_1/2_~17.5 min) > **IMB** (τ_1/2_~15.6 min) > **2IMB** (τ_1/2_~2.9 min). When both dye families are compared, a clear difference in photostability behavior emerges. Under identical irradiation power (75 mW/cm), **MB** derivatives (**MB**, **IMB**, **2IMB**) demonstrated substantially longer half-lives (minutes), whereas cyanine derivatives exhibited much faster decay (seconds). This pronounced contrast reflects intrinsic molecular differences between the phenothiazinium and polymethine scaffolds. While iodination in both series promoted intersystem crossing and enhanced ROS generation, its impact on photostability was more severe in the cyanines, causing rapid bleaching. Conversely, **MB** and its iodinated analogs maintained higher photochemical stability.

### 2.4. Quantum Yields of Singlet Oxygen and ROS Generation

Experimental conditions for measuring the quantum yields of singlet oxygen (Φ_Δ_) and ROS (φ_ROS_) generation, including the concentrations of the photosensitizer and scavenger (DPBF, SOSG, and H_2_DCFDA), as well as the light power density (irradiance) and illumination time, were optimized based on preliminary tests with varying parameters.

The singlet oxygen yields (Φ_Δ_) for cyanine dyes in saline and methanol were previously reported [21,28] but remeasured in this work in saline (Table 1) together with the new MB dye series to ensure that all experiments were performed under identical conditions. For both the cyanine and **MB** derivatives, Φ_Δ_ values were determined in saline using SOSG as the singlet oxygen trap, and in methanol or acetonitrile using DPBF as a singlet oxygen scavenger.

**Figure 3:**
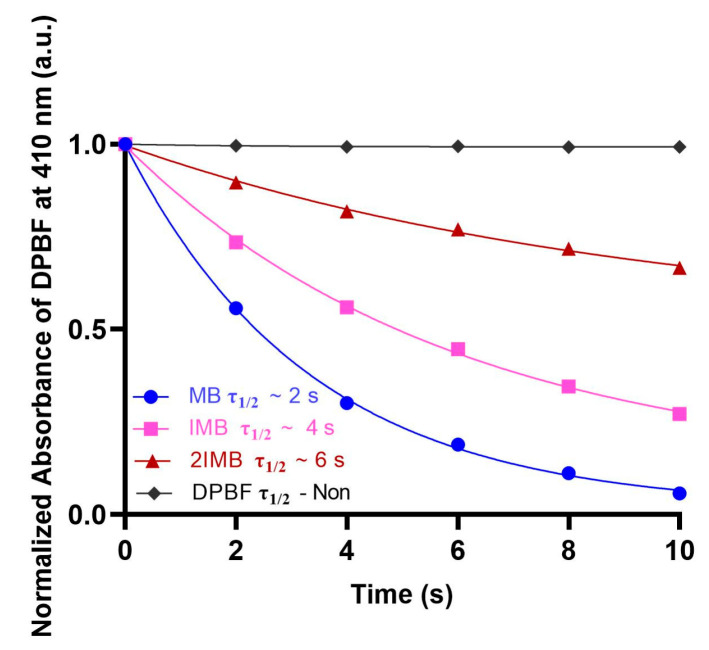
Kinetic curves for the Φ_Δ_ measurements of **MB** derivatives in acetonitrile. Normalized absorbances of DPBF (*A*~0.20) at 410 nm vs. light exposure time, when illuminated in a mixture with **MB**, **IMB**, and **2IMB** (*A*~0.25) in acetonitrile, and the corresponding half-lives (τ_1/2_). Irradiation using a 632-nm LED (1 mW/cm^2^). There is no detectable decrease in DPBF absorption either in the dark or upon light irradiation at the investigated light doses.

**Figure 4:**
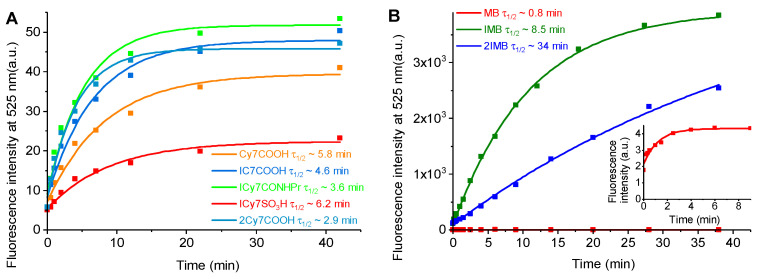
Kinetic curves for the Φ_Δ_ measurements of cyanines and MB derivatives in saline. Normalized fluorescence of SOSG (*A*~0.20) at 525 nm vs. light exposure time, when illuminated in a mixture with (**A**) cyanines (*A*~0.10) and (**B**) **MB**, **IMB**, **2IMB** (*A*~0.10) in saline, and the corresponding half-lives (τ_1/2_). Irradiation using a 30 W, 730 nm LED for cyanines and a 632 nm LED for MB derivatives. Irradiance was 1 mW/cm^2^.

Briefly, mixtures of dye and scavenger solutions were irradiated with a 730 nm LED (for cyanines) or a 632 nm LED (for MB derivatives) The increasing SOSG or H_2_DCFDA fluorescence or the decreasing DPBF absorbance was plotted against irradiation time and fitted with a monoexponential decay function (Figure 3 and Figure 4). The corresponding time-dependent spectra are shown in Figures S1–S3. The Φ_Δ_ values were calculated using Equation (2) with Cy7CO_2_H (Φ_Δ_ = 9.5% in saline) [28] and MB (Φ_Δ_ = 52% in acetonitrile) [40] as the references (Table 2).

To ensure that the measurements were completed within a practical time scale (longer than several seconds but shorter than several hours), irradiances of 100 mW/cm^2^ and 4 mW/cm^2^ were used for determining φ_ROS_ of cyanines and MB derivatives, respectively. For Φ_Δ_ measurements in saline, an irradiance of 40 mW/cm^2^ was applied to both dye series, while Φ_Δ_ values of the cyanines in methanol and MB dyes in acetonitrile were obtained at 1 mW/cm^2^. The pronounced difference in irradiance reflected the distinct singlet oxygen and ROS generation efficiencies of the cyanine and MB dyes, as well as the differing sensitivities of the DPBF, SOSG, and H_2_DCFDA scavengers toward singlet oxygen and other photoinduced reactive species.

**Figure 5:**
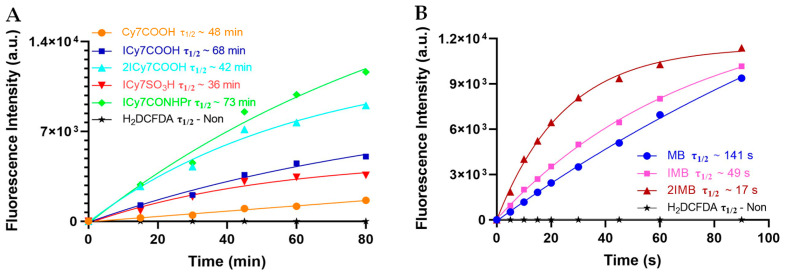
Kinetic curves for the φ_ROS_ measurements of cyanines and MB derivatives in saline. Fluorescence intensities of DCF, formed upon oxidation of H_2_DCFDA (*A* = 0.20) under 632 nm irradiation (1 mW/cm^2^) for **MB** derivatives (**A**) and 730 nm (100 mW/cm^2^) for cyanine dyes (**B**), as a function of irradiation time. The half-life (τ_1/2_) values of **Cy7CO_2_H** and H_2_DCFDA in (**A**) could not be determined within the experimental time range. There is no detectable increase in H_2_DCFDA fluorescence either in the dark or upon light irradiation at the investigated light doses.

The yields of ROS generation (φ_ROS_) for both dye classes were measured in saline by monitoring the increasing fluorescence intensity of DCF formed upon oxidation of H_2_DCFDA under 632 nm (4 mW/cm^2^) and 730 nm (100 mW/cm^2^) irradiation for **MB** derivatives and cyanines, respectively. The kinetic curves fitted with monoexponential functions are shown in Figure 5A,B, and the φ_ROS_ values are given in Table 1 and Table 2. The corresponding time-dependent spectra are shown in Figures S4 and S5.

Because the illumination time for the **MB** derivatives (up to 90 s at 4 mW/cm^2^, Figure 4A) was much shorter than the photobleaching half-lives (2.9–17.5 min at 75 mW/cm^2^, Figure 2), the photobleaching effect on the φ_ROS_ measurements was negligible.

The quantum yield of ROS generation (φ_ROS_) exhibits a clear increase upon iodination in both cyanines and **MB** dye series: **Cy7CO_2_H** << **ICy7CONHPr** < **ICy7CO_2_H** < **ICy7SO_3_H** < **2ICy7CO_2_H** (Table 1) and **MB** < **IMB** < **2IMB** (Table 2). In the cyanine dye series, φ_ROS_ depends not only on the number of iodine atoms but also on the solubilizing group. Thus, φ_ROS_ measured in saline for the mono-iodinated dyes increases in the following order: **ICy7CONHPr** < **ICy7CO_2_H** < **ICy7SO_3_H**, whereas in methanol the order changes to **ICy7CO_2_H** < **ICy7SO_3_H** < **ICy7CONHPr**.

The effects of iodines and solubilizing groups on the singlet oxygen generation yield (Φ_Δ_) in the cyanine dye series were thoroughly discussed in our previous work [28]. While iodination increases Φ_Δ_ measured in saline in the order **ICy7SO_3_H** < **Cy7CO_2_H** < **ICy7CO_2_H** < **ICy7CONHPr** < **2ICy7CO_2_H**, in methanol the order differs: **Cy7CO_2_H** < **ICy7CO_2_H** < **ICy7SO_3_H** < **2ICy7CO_2_H** < **ICy7CONHPr**. Remarkably, these orders are not the same as those observed for φ_ROS_, which indicates that there is no correlation between φ_ROS_ and Φ_Δ_. This non-synchronous change in φ_ROS_ and Φ_Δ_ indicates the generation of other ROS upon light irradiation, with their concentrations depending on the dye structure. Interestingly, the Φ_Δ_ values measured in aqueous saline are higher than those obtained in methanol.

An even stronger lack of correlation between φ_ROS_ and Φ_Δ_ in aqueous media was observed for the **MB** derivatives. While φ_ROS_ increases upon iodination (**MB** < **IMB** < **2IMB**), Φ_Δ_ conversely, decreases (**2IMB** > **IMB** > **MB**) in non-aqueous (acetonitrile) media and even more so in aqueous saline. These findings suggest that the introduction of iodine atoms into **MB** reduces singlet oxygen production, as measured with SOSG and DPBF, while enhancing the generation of other ROSs that are not detectable by the H_2_DCFDA scavenger. Thus, in MB derivatives, iodination facilitates the switching of the photodynamic mechanism from Type II to Type I.

Notably, the iodinated **MB** dyes are more efficient ROS generators than are iodinated cyanines. Because of the large difference in ROS generation efficiency between these two dye series, a direct quantitative comparison was not feasible. Accordingly, the ROS measurements required light doses of ~480 J/cm^2^ (at 100 mW/cm^2^) for the iodinated cyanines, whereas the **MB** derivatives required doses about three orders of magnitude lower (~0.4 J/cm^2^ at 3 mW/cm^2^). Nevertheless, the quantum yields of singlet oxygen generation for both dye series in saline were comparable: 8.3–18.9% for the cyanines and 0.6–60.5% for the **MB** derivatives. Φ_Δ_ was higher for the non-iodinated **MB** (60.5%) than for the non-iodinated cyanine **Cy7CO_2_H** (9.5%), whereas the opposite trend was observed for the mono-iodinated cyanines (8.3–14.1%) vs. **IMB** (5.6%) and the di-iodinated cyanine **2ICy7CO_2_H** (18.9%) vs. **2IMB** (0.6%). The solvent strongly impacts the Φ_Δ_ of cyanines, increasing from 1.1–2.5% in methanol to 8.3–18.9% in saline [28] (Table 1), whereas iodinated **MB** derivatives constitute 20–52% in acetonitrile and 0.6–60.5% in saline (Table 2).

### 2.5. Dye Uptake by Fungi

The uptake of a dye by fungal cells is the second most important parameter, after ROS and singlet oxygen generation efficiency, for determining dye phototoxicity. Dye uptake was measured using a spectrophotometric method [26]. Suspensions of *C*. *albicans* and *C*. *parapsilosis* (~10^6^ cells/mL, 1 mL per well) were incubated in saline with the tested dyes at three concentrations (0.05, 0.1, and 0.5 µM) for 30, 45, and 60 min at each concentration. Control wells with saline and dye but no cells were included to correct for any non-cellular dye loss or adsorption. Dye uptake was quantified as the complement of the ratio of fluorescence intensity in the supernatant to the initial dye solution. All the measurements were repeated for each concentration and incubation period. The dye concentration and incubation time had no statistically significant effect on uptake.

The uptake of cyanine and **MB** dyes by both fungal species moderately varied between 68 and 94% (Table 3). Only the sulfonated cyanine **ICy7SO_3_H**, characterized by increased hydrophilicity, showed notably reduced uptake (~27%). Iodination of **MB** tended to increase cellular accumulation, likely due to enhanced hydrophobicity. In the cyanine series, iodination had a slight effect on uptake, which remained consistently high except for the hydrophilic **ICy7SO_3_H**. These results suggest that differences in dye uptake between series and within series may have a minor to moderate influence on photocytotoxicity. **ICy7SO_3_H** is predicted to have reduced phototoxicity because of substantially lower fungal accumulation.

This analysis of dye uptake complements photophysical data to explain variations in phototoxic effects between dyes.

### 2.6. Photodynamic Versus AmpB Eradication of Candida Strains

Next, we evaluated the antifungal response of *C*. *albicans* and *C*. *parapsilosis* to PDT with cyanine- and **MB**-based PSs and compared it with that of **AmpB** treatment. Fungal mortality was assessed using colony-forming unit (CFU) counts and quantified through the median lethal concentration (LC_50_), which represents the concentration that kills 50% of the cell population.

For the estimation of the photodynamic effect, fungal suspensions in saline were incubated with dyes at concentrations of 0.05, 0.07, 0.1, 0.3, and 0.5 µM for 30 min, followed by 30 min of illumination. Light exposure was performed at a dose of 115 J/cm^2^ (irradiance 75 mW/cm^2^) using a 30 W LEDs at 730 nm for cyanines and 632 nm for **MB** derivatives. This dose was optimized, as increasing it further did not enhance cell killing, while reducing it decreased phototoxicity. After treatment, the fungi were cultured, and mortality (%) was quantified as the ratio between CFU post-treatment and CFU without light exposure.

**Figure 6:**
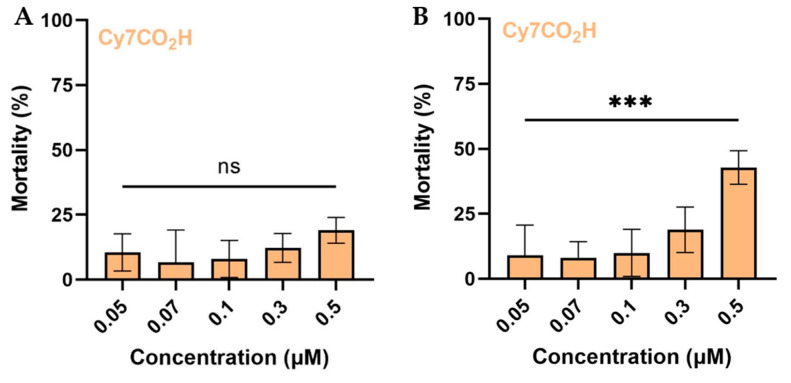
Effect of different concentrations of **Cy7CO_2_H** on the mortality rates of (**A**) *C*. *parapsilosis* and (**B**) *C*. *albicans* after PDT treatment. Statistical significance was determined using one-way ANOVA followed by Dunnett’s test, and the results were compared with those at the 0.5 µM concentration. Results are presented as the means ± SDs, with *n* = 4. Statistical significance is indicated as *** *p* < 0.001, ns = non-significant.

**Figure 7:**
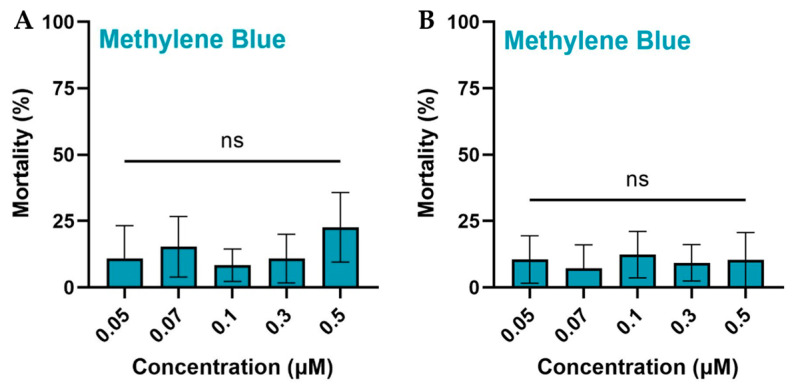
Effects of different concentrations of **MB** on the mortality rates of (**A**) *C*. *parapsilosis* and (**B**) *C*. *albicans* after PDT treatment. Statistical significance was determined using one-way ANOVA followed by Dunnett’s test, and the results were compared with those at the 0.5 µM concentration. Results are presented as the means ± SDs, with *n* = 4. Statistical significance is indicated as ns = non-significant.

**Figure 8:**
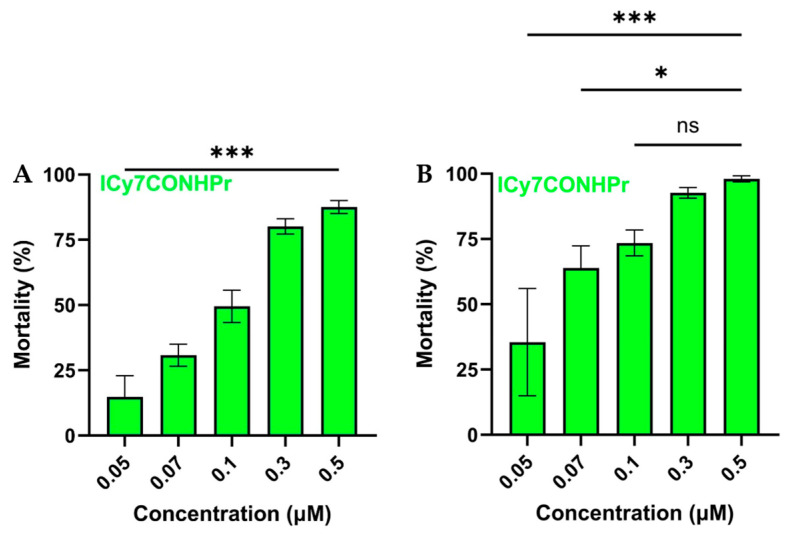
Effects of different concentrations of **ICy7CONHPr** on the mortality rates of (**A**) *C*. *parapsilosis* and (**B**) *C*. *albicans* after PDT treatment. Statistical significance was determined using one-way ANOVA followed by Dunnett’s test, and the results were compared with those at the 0.5 µM concentration. Results are presented as the means ± SDs, with *n* = 4. Statistical significance is indicated as *** *p* < 0.001, * *p* < 0.05, ns = non-significant.

**Figure 9:**
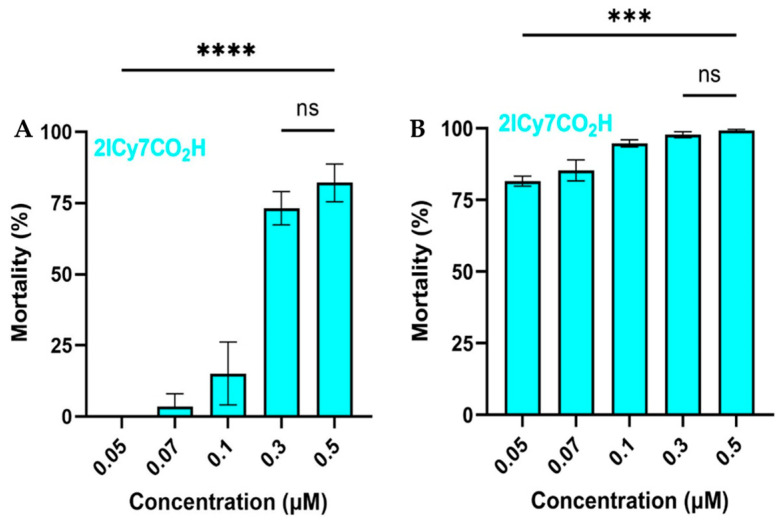
Effects of different concentrations of **2ICy7CO_2_H** on the mortality rates of (**A**) *C*. *parapsilosis* and (**B**) *C*. *albicans* after PDT treatment. Statistical significance was determined using one-way ANOVA followed by Dunnett’s test, and the results were compared with those at the 0.5 µM concentration. Results are presented as the means ± SDs, with *n* = 4. Statistical significance is indicated as **** *p* < 0.0001, *** *p* < 0.001, ns = non-significant.

**Figure 10:**
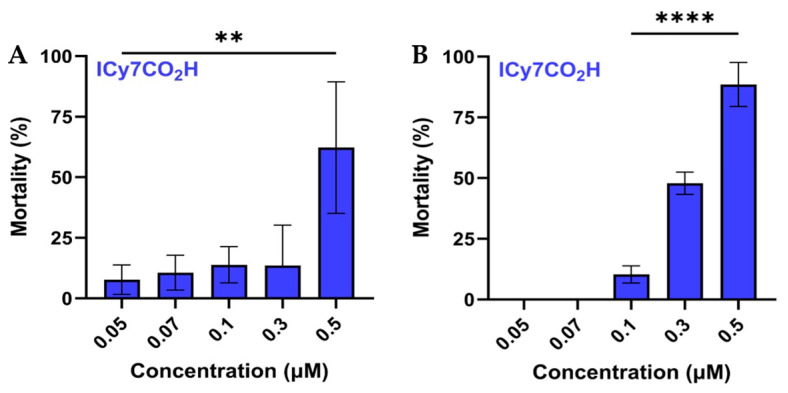
Effects of different concentrations of **ICy7CO_2_H** on the mortality rates of (**A**) *C*. *parapsilosis* and (**B**) *C*. *albicans* after PDT treatment. Statistical significance was determined using one-way ANOVA followed by Dunnett’s test, and the results were compared with those at the 0.5 µM concentration. Results are presented as the means ± SDs, with *n* = 4. Statistical significance is indicated as **** *p* < 0.0001, ** *p* < 0.01.

**Figure 11:**
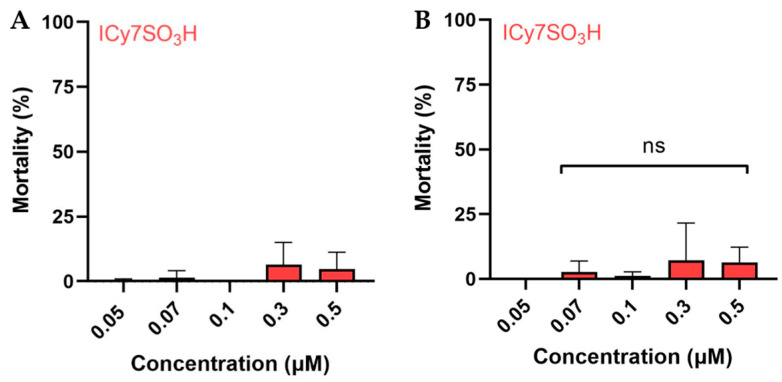
Effects of different concentrations of **ICy7SO_3_H** on the mortality rates of (**A**) *C*. *parapsilosis* and (**B**) *C*. *albicans* after PDT treatment. Statistical significance was determined using one-way ANOVA followed by Dunnett’s test, and the results were compared with those at the 0.5 µM concentration. Results are presented as the means ± SDs, with *n* = 4. Statistical significance is indicated as ns = non-significant.

**Figure 12:**
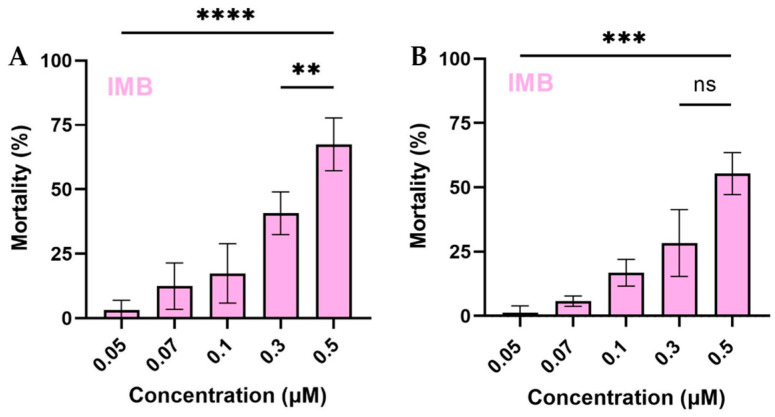
Effects of different concentrations of **IMB** on the mortality rates of (**A**) *C*. *parapsilosis* and (**B**) *C*. *albicans* after PDT treatment. Statistical significance was determined using one-way ANOVA followed by Dunnett’s test, and the results were compared with those at the 0.5 µM concentration. The results are presented as the means ± SDs, with *n* = 4. Statistical significance is indicated as **** *p* < 0.0001, *** *p* < 0.001, ** *p* < 0.01, ns = non-significant.

**Figure 13:**
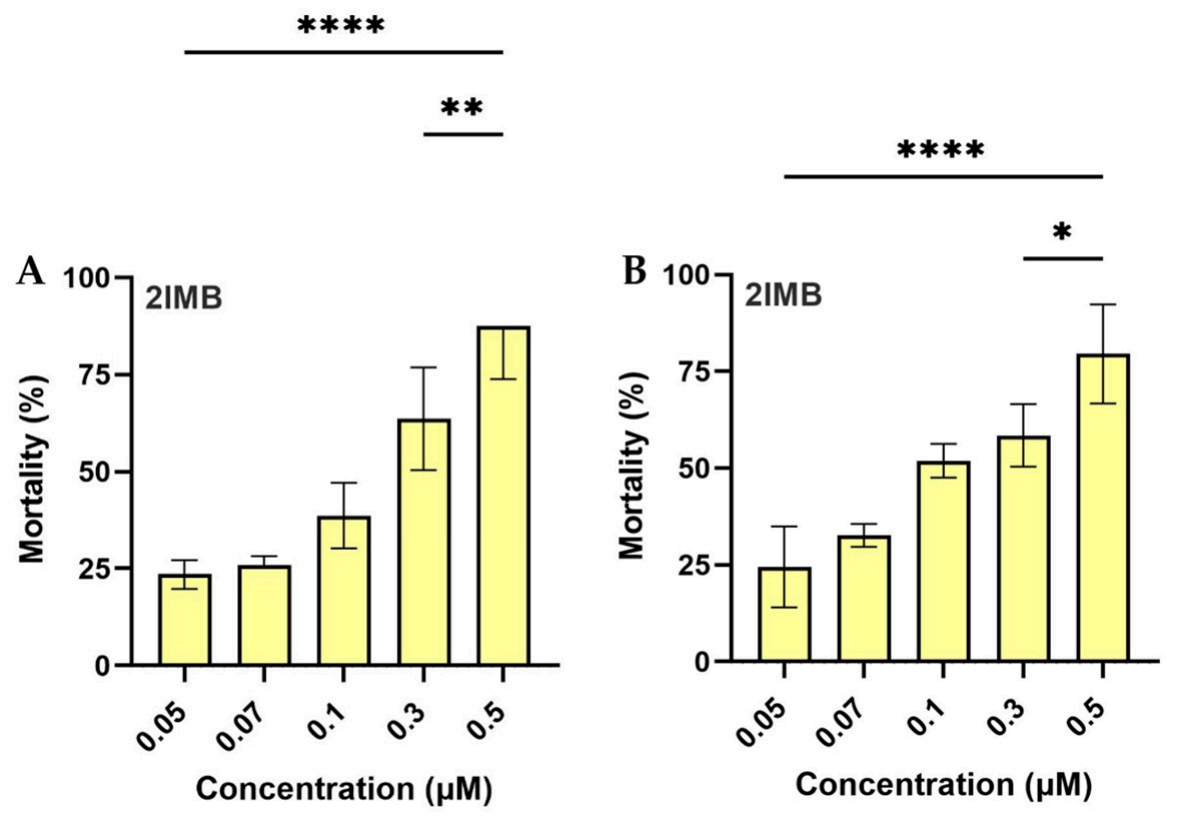
Effects of different concentrations of **2IMB** on the mortality rates of (**A**) *C*. *parapsilosis* and (**B**) *C*. *albicans* after PDT treatment. Statistical significance was determined using one-way ANOVA followed by Dunnett’s test, and the results were compared with those at the 0.5 µM concentration. Results are presented as the means ± SDs, with *n* = 4. Statistical significance is indicated as **** *p* < 0.0001, ** *p* < 0.01, * *p* < 0.05.

Diagrams demonstrating the mortality percentage vs. the dye structures and concentrations are presented in Figure 6, Figure 7, Figure 8, Figure 9, Figure 10, Figure 11, Figure 12 and Figure 13, while the LC_50_ values are shown in Table 4. For comparison, this table also contains the LC_50_ values for **AmpB** the treatments.

Control experiments confirmed that fungal inactivation was solely due to the PS photodynamic effect. Experiments in the dark revealed no fungal killing at any dye concentration. Additional controls with fungal samples incubated (i) in the dark without DMSO, (ii) in the dark with DMSO, and (iii) exposed to light with DMSO showed no effect on viability.

Upon light irradiation, both strains exhibited low sensitivity and failed to reach 50% mortality at any concentration, with non-iodinated dyes **Cy7CO_2_H** and **MB** reaching 0.5 µM (Figure 6 and Figure 7).

Conversely, PDT with iodinated cyanines achieved stronger killing, with sensitivity dependent on the dye structure. After treatment with monoiodo **ICy7CONHPr** (Figure 8A,B), both fungi exhibited photoinduced mortality. *C*. *albicans* showed higher mortality (Figure 8B) with LC_50_ at 0.06 µM (*C*. *albicans*) than *C*. *parapsilosis* (0.12 µM), with near-complete mortality at 0.5 µM in both strains.

Diiodo cyanine **2ICy7CO_2_H** (Figure 9A,B) exhibited even stronger eradication (compared to **ICy7CONHPr**) causing nearly complete mortality of *C*. *albicans* at 0.1 µM but killing of *C*. *parapsilosis* by ~15%. The LC_50_ values for **2ICy7CO_2_H** were 0. 21 µM (*C*. *parapsilosis*) and 0.020 µM (*C*. *albicans*).

Compared to **ICy7CONHPr** and **2ICy7CO_2_H**, the carboxy-derivatized mono-iodinated cyanine **ICy7CO_2_H** (Figure 10) exhibited reduced phototoxicity against both fungal species. Significant efficacy was observed only at 0.5 µM, with LC_50_ values of 0.45 µM for *C*. *parapsilosis* and 0.30 µM for *C*. *albicans*.

Sulfonated **ICy7SO_3_H** lacked statistically significant phototoxicity (Figure 11), which is consistent with its low fungal uptake.

While **MB** exhibits very low phototoxicity, iodinated **IMB** (Figure 12) and especially diiodinated **2IMB** (Figure 13) dyes show markedly increased phototoxicity. **2IMB** has a photokilling effect (~85% mortality at *c*_Dye_ = 0.5 µM) on *C*. *parapsilosis* comparable to that of the iodinated cyanine **ICy7CONHPr**. However, we did not achieve complete eradication of *C*. *albicans* with iodinated **MB** dyes, even at the maximal concentration tested (0.5 µM), whereas the iodinated cyanines **ICy7CONHPr** (Figure 8B) and **2ICy7CO_2_H** (Figure 9B) caused nearly 100% cell death.

The photokilling efficacy of **2IMB**, estimated by the LC_50_, was approximately 2.5-fold higher for *C*. *albicans* and 1.5-fold higher for *C*. *parapsilosis* than for **IMB** (Table 4). The LC_50_ values for **IMB** (0.45 µM for *C*. *albicans* and 0.34 µM for *C*. *parapsilosis*) and **2IMB** (0.18 µM for *C*. *albicans* and 0.23 µM for *C*. *parapsilosis*) indicated that both dyes were less efficient PSs than were the mono- and di-iodinated cyanines, except for **ICy7CO_2_H**, which was less effective than **2IMB**. The strongest photokilling effect against both fungal species was observed for diiodinated **2ICy7CO_2_H** and monoiodinated **ICy7CONHPr**. In *C*. *parapsilosis*, **2IMB** had an effect similar to that of **2ICy7CO_2_H**, although its efficacy was approximately half that of **ICy7CONHPr**.

**Figure 14:**
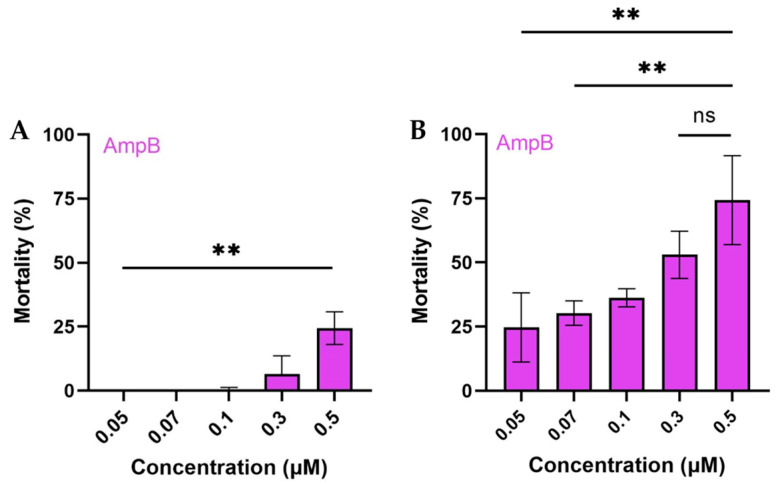
Effects of different concentrations of **AmpB** on the mortality rates of (**A**) *C*. *parapsilosis* and (**B**) *C*. *albicans*. Statistical significance was determined using one-way ANOVA followed by Dunnett’s test, and the results were compared with those at the 0.5 µM concentration. Results are presented as the means ± SDs, with *n* = 4. Statistical significance is indicated as ** *p* < 0.01, ns = non-significant.

The obtained phototoxicity data for the PSs were compared with those for conventional therapy using **AmpB** applied at the same concentrations (0.05, 0.07, 0.1, 0.3, and 0.5 μM). Figure 14 shows that the highest tested concentration of 0.5 μM **AmpB** induced only approximately 25% mortality of *C*. *parapsilosis*, which is considered insufficient for clinical treatment. Owing to this limited efficacy, no LC_50_ value could be determined for **AmpB** against *C*. *parapsilosis* within the tested concentration range.

In contrast, **AmpB** was more effective against *C*. *albicans*, resulting in approximately 75% mortality at 0.5 μM (LC_50_ = 0.20 μM, Table 4). The **AmpB** concentration needed to achieve 25% mortality in *C*. *parapsilosis* was tenfold higher (0.5 µM) than that required for the same effect in *C*. *albicans* (0.05 µM). Nonetheless, the 75% mortality observed with **AmpB** in *C*. *albicans* was still inferior to that of PDT with the iodinated cyanines **ICy7CONHPr** (Figure 8B) and **2ICy7CO_2_H** (Figure 9B), which presented LC_50_ values of 0.06 μM and 0.02 μM, respectively, and almost completely eradicated this strain even at 0.3 μM.

## 3. Discussion

In this study, the efficacy of PDT based on photosensitizers from the cyanine and **MB** families was evaluated against *C*. *albicans* and *C*. *parapsilosis*. These findings reveal species-dependent susceptibility and considerable differences in compound performance. **AmpB**, the first-line antifungal agent, was moderately effective against *C*. *albicans*, whereas significantly higher concentrations were required to achieve a similar effect against *C*. *parapsilosis*. This observation is in agreement with previous reports [41] and underscores the need for alternative therapeutic strategies. Therefore, we considered photosensitizers that exhibited measurable LC_50_ values for *C*. *parapsilosis* within the tested range as effective against this species. With respect to *C*. *albicans*, we assumed that photosensitizers with LC_50_ values lower than those of **AmpB** may offer a therapeutic advantage.

The antifungal activity of cyanine derivatives upon light irradiation demonstrated a clear dependence on both the presence of iodine atoms and the linker functional group. The non-iodinated photosensitizer **Cy7CO_2_H** did not have a measurable LC_50_, whereas the introduction of one iodine atom, such as **ICy7CO_2_H** markedly enhanced antifungal activity. The di-iodinated dye **2ICy7CO_2_H** achieved nearly complete killing at the highest concentration tested (0.5 µM) and exhibited the lowest LC_50_ values in both species. Similar results were obtained for **ICy7CONHPr**, which has a single iodine atom but contains an amide rather than a carboxylic group at its linker terminus, a modification likely affecting solubility, membrane permeability, and intracellular localization [28]. In contrast, iodinated **ICy7SO_3_H** displayed weak antifungal activity due to its pure uptake associated with reduced lipophilicity and repulsion between negatively charged dye molecules and negatively charged phospholipids in fungal membranes.

Within the **MB** family, the parent **MB** dye exhibited limited antifungal efficacy in both species, which is consistent with earlier reports [42]. As expected, its iodinated derivatives, **IMB** and **2IMB**, demonstrated markedly enhanced phototoxic activity. Interestingly, **IMB** showed the opposite species sensitivity pattern. *C*. *parapsilosis* was slightly more susceptible than *C*. *albicans* possibly due to differences in its intracellular localization or metabolism. The di-iodinated derivative **2IMB** further amplified the effect, achieving over 75% killing in both species at 0.5 µM. In addition to their high efficacy, **IMB** and **2IMB** offer practical advantages: **MB** is inexpensive and widely available, and iodination requires only a single synthetic step, unlike the more complex and costly synthesis of iodocyanine derivatives.

The enhanced antifungal activity following iodination can be explained by the heavy-atom effect. The incorporation of heavy atoms such as iodine increases intersystem crossing (ISC) through spin–orbit coupling, promoting triplet-state formation and the subsequent generation of reactive oxygen species (ROS), including singlet oxygen. In this study, **ICy7CO_2_H** and **IMB**, each bearing one iodine atom, presented similar LC_50_ values (0.30 µM and 0.45 µM, respectively) in *C*. *albicans*, whereas their non-iodinated counterparts (**Cy7CO_2_H** and **MB**) exhibited weak or undetectable activity. The difference became even more pronounced when mono- and di-iodinated sensitizers were compared: **2ICy7CO_2_H** was approximately 15-fold more potent than **ICy7CO_2_H**, and **2IMB** was almost twice as effective as **IMB**.

The quantum yields of singlet oxygen generation (Φ_Δ_) and overall ROS formation (φ_ROS_) were measured to better understand the photochemical contribution to antifungal activity. These parameters were determined using specific scavengers for each photosensitizing mechanism: DPBF and SOSG, preferably specific for singlet oxygen (Type II photosensitization) [43], and less specific H_2_DCFDA for radical ROS (Type I photosensitization) [44]. Typically, both Type I and Type II mechanisms can operate concurrently, with their relative contributions depending on factors such as the oxygen concentration, chemical structure of the PS, and microenvironment [45].

Analysis of the cyanine derivatives indicated that iodination affects the two photochemical pathways differently. An increase in the number of iodine atoms led to higher singlet oxygen yields and an even more pronounced enhancement in overall ROS generation. Owing to their different solubilizing properties, the terminal functional group at the linker also strongly influences photochemical behavior and cell uptake. The monoiodinated **ICy7CONHPr**, which displayed antifungal activity similar to that of the diiodinated **2ICy7CO_2_H**, produced relatively low levels of ROS but elevated levels of singlet oxygen. Conversely, despite its low antifungal activity, **ICy7SO_3_H** presented relatively high Φ_Δ_ and φ_ROS_ values in aqueous media (e.g., relative to **IMB** and **2IMB**). However, as mentioned above, its low membrane permeability (uptake) limits its phototoxicity. These observations suggest that iodination and functional group modification can differentially alter ROS generation and antifungal efficacy.

For the **MB** derivatives, the opposite trend was observed: Φ_Δ_ decreased with iodination (**MB** > **IMB** > **2IMB**), whereas overall ROS generation increased (**MB** < **IMB** < **2IMB**). This pattern reflects a mechanistic shift from a singlet-oxygen-dominated Type II pathway in **MB** to a radical-mediated Type I photosensitization process in the iodinated derivatives. Mechanistically, the iodine atoms introduced into the MB derivatives play a crucial role in this photodynamic switch. In addition to enhancing intersystem crossing (ISC) through the heavy-atom effect, these iodine atoms can directly participate in electron-transfer reactions from the dye triplet state to molecular oxygen or other substrates. This electron-transfer pathway promotes the formation of radical species such as superoxide and hydroxyl radicals, which are characteristic ROSs involved in Type I photosensitization, in contrast to singlet oxygen generated via energy transfer in Type II processes. Moreover, iodinated MB derivatives may generate reactive iodine species via redox cycling, further amplifying radical ROS production. Therefore, the iodine atoms themselves facilitate enhanced radical generation through electron transfer, inducing a mechanistic shift from predominantly singlet oxygen-based Type II photochemistry towards Type I radical pathways.

Furthermore, a sharp decline in the fluorescence quantum yield (Φ_F_) was observed with iodination: while **MB** exhibited strong fluorescence, **IMB** and **2IMB** were almost non-fluorescent, with the emission intensity decreasing by orders of magnitude as the number of iodine atoms increased. This finding supports the hypothesis that iodination promotes deactivation of the singlet-excited state via ISC to the triplet state, thereby explaining both the mechanistic shift and the higher antifungal efficiency of **2IMB** despite its relatively low Φ_Δ_ value. The observed decrease in fluorescence quantum yield with increasing iodination can be explained by the absorption spectra, which show that the addition of iodine atoms enhances the shoulder on the blue side, indicating the formation of *H*-aggregates. In most cases, such *H*-aggregates lead to a pronounced reduction in fluorescence intensity due to excitonic interactions and quenching effects [23].

Photostability experiments revealed that **MB** was more stable in aqueous solution than its iodinated counterparts, with **2IMB** being the least stable. The reduced stability of the iodinated derivatives may result from an increased ISC rate and triplet state lifetime (associated with enhanced ROS production), since dissolved oxygen facilitates photobleaching through singlet-oxygen formation from the triplet state [22,23]. In contrast, iodocyanines exhibited relatively good photochemical stability compared with iodo-**MB**s. Biologically, greater stability may prolong activity; however, when ROS production is rapid and intense, as observed for **IMB**, reduced stability may be compensated by strong antifungal efficacy within short time frames.

In addition to photochemical properties, cellular uptake plays a major role in determining antifungal efficacy. Iodocyanines showed high cellular uptake in both fungal species except for **ICy7SO_3_H**, which exhibited much lower penetration. The uptake of PSs was, in general, only negligibly higher for *C*. *albicans* than for *C*. *parapsilosis*; therefore, the increased mortality of *C*. *albicans* upon treatment with PSs and **AmpB** is more likely due to the increased sensitivity of this strain.

Although photosensitizer uptake was similar between *C*. *albicans* and *C*. *parapsilosis*, the greater PDT susceptibility of *C*. *albicans* may reflect intrinsic biological differences between the species. Previous studies have shown that *C*. *albicans* forms a thicker and more complex biofilm than *C*. *parapsilosis* [46], and that the two species also differ in cell-wall mannan organization and lipid homeostasis systems [47]. In *C*. *parapsilosis*, the stearoyl-CoA desaturase Ole1 plays a central role in the synthesis of unsaturated fatty acids (UFAs), which are essential to maintaining membrane fluidity, stability, and appropriate stress responses [48]. This enhanced ability to regulate and preserve membrane lipid composition may provide *C*. *parapsilosis* with an advantage in coping with photodynamically induced membrane damage, contributing to the higher resilience observed under the PDT conditions tested. Nevertheless, these mechanisms were not directly examined in the present study and warrant further investigation.

Overall, these findings suggest that iodinated cyanines represent a more promising structure for developing highly phototoxic PSs against both *C*. *albicans* and *C*. *parapsilosis*, while iodinated **MB** derivatives have comparable efficiency in *C*. *parapsilosis*. An additional advantage of iodinated cyanines over **MB** derivatives is their strong fluorescence, which enables real-time monitoring in vivo. There is a high probability that further improvement of iodinated cyanines can be achieved by combining the structural benefits of **ICy7CONHPr** and **2ICy7CO_2_H**, specifically by derivatizing the carboxylic group in **2ICy7CO_2_H** with propylamide. An important advantage of iodinated **MB** derivatives over cyanines is their synthetic accessibility and substantially lower production cost.

The cytotoxicity of photosensitizers arises from a combination of factors, including singlet oxygen generation, the type and yield of free radicals, the membrane permeability of these RSs, their intracellular localization, and their accumulation within particular organelles such as mitochondria, lysosomes, and the rough endoplasmic reticulum (RER).

Future research should focus on elucidating the precise biological mechanisms underlying the antifungal efficacy of photosensitizers, including singlet oxygen and ROS generation, membrane permeability of reactive species, intracellular localization, and accumulation within key organelles such as mitochondria and lysosomes. Detailed studies on the cellular uptake pathways and subcellular targeting will enhance the understanding of PS functionality and optimize design.

Furthermore, validation of these photosensitizers under relevant in vivo conditions is essential for assessing their therapeutic potential and safety profiles in complex biological systems. Optimizing treatment parameters, including photosensitizer concentrations, light dosimetry, and exposure timing, is critical for maximizing effectiveness while minimizing side effects.

Advances in PS structural optimization, particularly combining beneficial features from cyanine derivatives (e.g., **ICy7CONHPr** and **2ICy7CO_2_H**), could improve efficacy and selectivity. The integration of real-time fluorescence imaging to monitor PS delivery and localization in vivo offers opportunities for personalized therapy and treatment monitoring.

Finally, exploration of combination therapies, such as coupling PDT with conventional antifungals or immune modulators, may enhance the efficacy against resistant fungal strains and biofilms, addressing current challenges in antifungal treatment.

## 4. Materials and Methods

### 4.1. General

All chemicals were obtained from Alfa Aesar Israel and Sigma-Aldrich, Israel. The solvents were sourced from Bio-Lab Israel and used without further purification. The progress of the chemical reactions was monitored using thin-layer chromatography (TLC) on Silica gel 60 F-254 plates (Merck, Israel) and by LC/MS analysis. LC/MS measurements were performed with an Agilent Technologies 1260 Infinity LC system coupled to a 6120 quadrupole mass spectrometer (MS) with an Agilent Zorbax SB-C18 column (1.8 µm, 2.1 mm × 50 mm) maintained at 50 °C. The mobile phase consisted of water and acetonitrile (ACN) with 0.1% formic acid. High-resolution mass spectrometry (HRMS) was performed in positive electrospray ionization (ESI) mode using an Agilent 6550 iFunnel Q-TOF LC/MS detector. Proton (^1^H) and carbon (^13^C) NMR spectra were recorded on a Bruker Avance III HD spectrometer operating at 400 MHz (^1^H) and 100 MHz (^13^C) and equipped with a BBO probe and a *Z*-axis gradient coil.

### 4.2. Synthesis of Photosensitizers

#### 4.2.1. Cyanines

While specific synthetic procedures for cyanines are not detailed here, they follow conventional condensing agent-mediated methods, as described in previous studies [21,28]. These reactions involve a one-pot sequential reaction of *N*-[5-(phenylamino)-2,4-pentadienylidene]aniline with the first indolenine molecule in acetic anhydride to form a corresponding *N*-phenylacetamide derivative, which subsequently reacts with the second indolenine in the presence of pyridine.

#### 4.2.2. Iodinated Methylene Blue (**IMB**)

*p*-Toluenesulfonic acid (pTSA, 26.9 mg, 0.156 mmol) and *N*-iodosuccinimide (NIS, 28 mg, 0.125 mmol) were added to a suspension of **MB** (50 mg, 0.156 mmol) in acetonitrile and stirred at RT for 1 h. The obtained **IMB** was column-purified (Silica gel) using a dichloromethane (DCM)–methanol (MeOH) gradient, starting from 3% MeOH and increasing to 10% MeOH. ^1^H NMR (400 MHz, DMSO-d6, ppm): δ 8.14 (s, 1H), 7.47 (d, J = 8.0 Hz, 2H), 7.10 (d, J = 8.4 Hz, 2H), 3.34 (s, 12H). ^13^C NMR (100 MHz, DMSO-d6, ppm): δ 159.7, 157.7, 152.0, 151.7, 148.1, 144.3, 138.3, 132.0, 129.4, 127.6, 123.2, 119.8, 43.3, 38.3. HRMS *m*/*z* (ESI^+^) C_16_H_17_IN_3_S^+^ calculated [M]^+^ 410.0187, found *m*/*z*: 410.0200.

#### 4.2.3. Diiodinated Methylene Blue (**2IMB**)

In a suspension of **MB** (50 mg, 0.156 mmol) in acetonitrile, 4-toluenesulfonic acid (26.9 mg, 0.156 mmol) and *N*-iodosuccinimide (NIS, 70 mg, 0.312 mmol) were added. The mixture was stirred at RT for 2 h. The resulting diiodinated product, **2IMB**, was purified by column chromatography on Silica gel using a dichloromethane (DCM)–methanol (MeOH) gradient, starting from 3% MeOH and increasing to 10% MeOH. ^1^H NMR (400 MHz, chloroform-d, ppm): δ 7.51 (d, J = 8.2 Hz, 2H), 7.06 (d, J = 8.6 Hz, 2H), 2.77 (s, 12H). ^13^C NMR (100 MHz, Chloroform-d, ppm): δ 158.8, 150.5, 142.1, 131.6, 128.3, 119.2, 45.4. HRMS *m*/*z* (ESI^+^) C_16_H_16_I_2_N_3_S^+^ calculated [M]^+^ 535.9154, found *m*/*z*: 535.9156.

### 4.3. Absorption and Fluorescence Measurements

The absorption and emission characteristics of the cyanine dyes were obtained from our previous studies [21,28]. For the **MB** derivatives, absorption spectra were recorded using a Jasco V-730 UV–Vis spectrophotometer, and fluorescence spectra were measured on an Edinburgh FS5 spectrofluorometer. Measurements were conducted at 25 °C in a standard 1-cm quartz cuvette at an excitation wavelength (λ*) of 620 nm.

To determine the absolute fluorescence quantum yields (Φ_F_), the integrated relative intensities of the dye were measured vs. the commercially available **MB** as the reference (Φ_F_ = 2%), and the quantum yields were calculated according to Equation (1).

The absolute fluorescence quantum yields (Φ_F_) were determined by comparing the integrated fluorescence intensities of the sample dyes with a commercially available **MB** reference (Φ_F_ = 2%) [39]. The quantum yields were calculated according to Equation (1).

Φ_F_ = Φ_F,Ref_ × (*F*/*F*_Ref_) × (*A*_Ref_/*A*), (1)

where Φ_F,Ref_ is the quantum yield of the reference, *F*_Ref_ and *F* are the integrated emission intensities (i.e., areas under the emission spectra) of the emission spectra of the reference and sample dyes, respectively, and *A*_Ref_ and *A* are the absorbances of the reference and sample dyes at the excitation wavelength.

### 4.4. Photostability

Stock solutions of the dyes were prepared in DMSO and diluted in 0.9% physiological saline to achieve final absorbances of approximately 0.03 at 632 nm. All the solutions contained 0.7% DMSO. The solutions were filtered using syringe filters and transferred to standard 1-cm quartz cuvettes. Light irradiation was performed using a 30 W LED equipped with a 60° lens to disperse light uniformly. The distance between the light source and the samples for **MB** derivatives was 8.0 cm (irradiated at 632 nm). The light power density was maintained at 75 mW/cm^2^.

Absorption spectra were recorded over predefined time intervals. The normalized absorbance at the absorption maximum of the dye was plotted as a function of illumination time. These data were fitted with a monoexponential decay function to estimate photostability via the decay half-life (τ_1/2_). All measurements were performed in triplicate, and average values were reported.

### 4.5. Quantum Yields of Singlet Oxygen Generation

The singlet oxygen quantum yields (Φ_Δ_) of the cyanine dyes in methanol were taken from our previous publications [21,28], whereas those in saline were measured in this work. For the **MB** derivatives, Φ_Δ_ was measured in acetonitrile following the method described in [40]. Solutions containing 1,3-diphenylisobenzofuran (DPBF, absorbance ~0.20 at 410 nm) and the MB-derivatized dye under examination (absorbance ~0.25 at an excitation wavelength of 632 nm) were prepared in acetonitrile. Samples of **MB**, **IMB**, and **2IMB** were placed in standard 1-cm quartz cuvettes and irradiated at a 50 cm distance using a 632 nm, 30 W LED with a 60° lens for light dispersion at a light power density of 1 mW/cm^2^.

Absorption spectra were collected over time, and DPBF absorbances at 410 nm were corrected for dye absorbance overlap measured under identical conditions without DPBF. The corrected DPBF absorbance data were normalized, plotted against irradiation time, and fitted with first-order exponential decay functions. The Φ_Δ_ values were calculated using Equation (2), with **MB** (Φ_Δ_ = 52% in acetonitrile [40]) as the reference.

Φ_Δ_ = Φ_Δ,Ref_ × (*k/k*_Ref_) × (*A*_Ref_/*A*), (2)

where Φ_Δ,Ref_ is the singlet oxygen quantum yield of the reference dye (**MB**), *k* and *k*_Ref_ are the DPBF degradation rates derived from the kinetic fits for the sample and reference dyes, respectively, and *A* and *A*_Ref_ are the absorbances of the sample and reference dyes at the excitation wavelength (632 nm), respectively.

The Φ_Δ_ values for the cyanine and **MB** dye series in saline were measured using a procedure similar to that described above for **MB** in acetonitrile. Solutions containing SOSG (5 mM) and cyanine or **MB**-derivatized dye in saline with 15% DMSO (*A* = 0.10 at 730 nm and 630 nm, respectively) were irradiated using a 730 nm LED for cyanine and a 632 nm LED for **MB** dyes (30 W, irradiance 40 mW/cm^2^). The fluorescence intensities at 525 nm (SOSG emission) were plotted as a function of irradiation time and fitted with first-order exponential decay functions. The Φ_Δ_ values were calculated using Equation (2), with **Cy7CO_2_H** (Φ_Δ_ = 9.5% [28]) as the reference.

Each experiment involving the Φ_Δ_ measurements was performed in triplicate, and the average Φ_Δ_ values were reported.

### 4.6. Quantum Yield of ROS Generation

The quantum yields of reactive oxygen species (ROS) generation (φ_ROS_) were determined using the fluorogenic probe 2′,7′-dichlorodihydrofluorescein diacetate (H_2_DCFDA, Lumiprobe, Batch 9GC7V). Upon light irradiation in the presence of photosensitizers, H_2_DCFDA is oxidized to fluorescent dichlorofluorescein (DCF).

Stock solutions of PSs and H_2_DCFDA were prepared in a minimal volume of methanol and subsequently diluted with saline. The final PS concentrations were adjusted to yield an absorbance of approximately 0.15 at the excitation wavelength (632 nm for MB dyes and 730 nm for cyanines). The samples containing H_2_DCFDA (absorbance ~0.20) and the PS under investigation (absorbance ~0.15) were irradiated with a 632 nm, 30 W LED positioned 30 cm from the sample, with a light power density of 4 mW/cm^2^ for the MB derivatives. The cyanine dyes were irradiated with a 730 nm, 30 W LED at 5 cm, delivering a light power density of 100 mW/cm^2^.

Fluorescence spectra were recorded over time with excitation at 495 nm (appropriate for DCF excitation). Kinetic curves representing the fluorescence intensity of DCF at 525 nm versus illumination time were fitted to monoexponential functions. The φ_ROS_ values were calculated using Equation (3):

φ_ROS_ = φ_ROS,Ref_ × (*k/k*_Ref_) × (*A*_Ref_/*A*), (3)

where φ_ROS,Ref_ is the ROS quantum yield of the reference dye (either **MB** or **ICy7CO_2_H**), *k* and *k*_Ref_ are the rates of DCF fluorescence increase obtained from the fittings for the sample and reference dyes, respectively, and *A* and *A*_Ref_ denote absorbances of the sample and reference dyes at their excitation wavelengths (632 nm for MB dyes, 730 nm for cyanines).

Control experiments, which were conducted without photosensitizers under identical irradiation conditions, confirmed that the minimal contribution from the intrinsic fluorescence increase in H_2_DCFDA.

All the measurements were performed in triplicate, and the averaged φ_ROS_ values are reported.

### 4.7. Fungal Strains

Two fungal strains, *C*. *albicans* (ATCC 24433) and *C*. *parapsilosis* (ATCC 22019), which were supplied by Beit HaEmek (Israel), were used in this study.

### 4.8. Preparation and Growth of Fungi

*C*. *albicans* and *C*. *parapsilosis* strains were cultured on Difco™ Yeast Mold (YM) agar plates (BD, Franklin Lakes, NJ, USA) and incubated at 37 ± 1 °C for 48 h. Subsequently, 2–3 yeast colonies were inoculated into Difco™ YM broth (BD) and incubated with shaking at 200 rpm for 24 h at 37 ± 1 °C.

The resulting fungal suspension was diluted with physiological saline (0.90% *w*/*v* NaCl in distilled water) to achieve optical densities of *A*_530_ = 0.10 ± 0.02 for *C*. *albicans* and *A*_490_ = 0.10 ± 0.02 for *C*. *parapsilosis*. Absorbance measurements were performed using a Genesys 10S UV–Vis spectrophotometer (Thermo Fisher, Waltham, MA, USA). In accordance with the 0.5 McFarland standard method, these absorbance values correspond to a cell concentration of approximately 1 × 10^6^–3 × 10^6^ cells/mL.

### 4.9. Dye Uptake by Fungal Cells

Suspensions of *C*. *albicans* and *C*. *parapsilosis* (~10^6^ cells/mL, 1 mL per well) were incubated in physiological saline containing the test dyes at three concentrations (0.05, 0.1, and 0.5 µM) at 25 °C in the dark for predetermined time intervals (30, 45, and 60 min). Following incubation, the samples were centrifuged at 13,400 rpm for 12 min to pellet the fungal cells. The supernatants containing unbound dye were carefully collected.

The fluorescence intensities of the supernatants (*F*_supernatant_) and corresponding reference dye solutions without fungal cells (*F*_Dye_) were measured at emission maxima specific to each dye using an Infinite M200 ELISA microplate reader (Tecan). Dye uptake was quantified as the complement of the ratio of fluorescence intensity in the supernatant to that in the initial dye solution, expressed as a percentage according to Equation (4).

%*Uptake* = [1 − (*F*_supernatant_/*F*_Dye_)] × 100% (4)

All measurements were performed in triplicate for each dye concentration and incubation period, and average values were reported.

### 4.10. Photodynamic Treatment of C. albicans and C. parapsilosis

All preparatory procedures involving photosensitizers were performed in the dark to prevent premature activation and photobleaching. Stock solutions of cyanines and **MB** dyes were prepared in DMSO at concentrations of 0.05, 0.07, 0.1, 0.3, and 0.5 µM (confirmed spectrophotometrically). Subsequently, 10 µL of each dye solution was added to 1 mL of fungal suspension (10^3^ cells/mL) in 0.9% saline in a Falcon^®^ 48-well polystyrene clear flat-bottom plate at a final DMSO concentration of 1% for all the samples.

The samples were incubated in the dark at RT for 30 min, followed by exposure to light under continuous shaking for an additional 30 min. Control samples were kept in the dark. Light exposure was carried out using 30 W LEDs emitting at 730 nm for cyanines and at 632 nm for **MB** derivatives, each equipped with a 60° lens for light dispersion. The light source was positioned at 6.5 cm (cyanines) and 8.0 cm (**MB** dyes) from the samples, delivering an irradiance of 75 mW/cm^2^ and a light dose of 115 J/cm^2^.

Post-irradiation, 100 µL aliquots of each sample were spread onto YM agar plates via a Drigalski spreader and incubated at 37 ± 1 °C for 24 h (*C*. *albicans*) or 48 h (*C*. *parapsilosis*). Colony-forming units (CFUs) were quantified using a Scan 500 colony counter (Interscience, Saint-Nom-la-Bretèche, France). Dark toxicity was assessed in parallel by conducting identical procedures without light exposure.

The control groups included fungal samples incubated under the following conditions: (i) in the dark without DMSO, (ii) in the dark with 1% DMSO, and (iii) exposed to light with 1% DMSO. All experiments were performed in duplicate and repeated three times on different days to ensure reproducibility.

Fungal mortality was evaluated based on CFU counts and quantified by calculating the median lethal concentration (LC_50_) required to kill 50% of the cell population. Each experiment was performed in triplicate, and average values were reported.

### 4.11. Statistical Analysis

The data are presented as the means ± SDs from at least three independent experiments (*n* = 3). Statistical comparisons were performed using one-way analysis of variance (ANOVA), followed by Dunnett’s multiple comparisons test to compare each treatment group against the control. All the analyses were conducted via GraphPad Prism version 10.0.1. Statistical significance was determined based on adjusted *p*-values from Dunnett’s test, which accounts for multiple comparisons while controlling for the family-wise error rate.

## 5. Conclusions

This study demonstrated the enhanced photodynamic antifungal efficacy of iodinated cyanine and methylene blue photosensitizers (PSs) against *C*. *albicans* and **AmpB**-resistant *C*. *parapsilosis* strains. The molecular structure of PSs influences their antifungal selectivity toward different fungal species. Iodinated cyanines **ICy7CO_2_H**, **ICy7CONHPr**, and **2ICy7CO_2_H** exhibited superior efficacy against both *C*. *albicans* and *C*. *parapsilosis*, whereas iodinated methylene blue derivatives **IMB** and **2IMB** showed particularly potent activity against the clinically challenging *C*. *parapsilosis*, with less selectivity toward *C*. *albicans*.

Iodination significantly enhanced the rate of reactive species generation and photodynamic cytotoxicity via the heavy-atom effect, which facilitates intersystem crossing and triplet-state formation. However, while reactive oxygen species (φ_ROS_) generation increased upon iodination for both cyanine and **MB** dyes, singlet oxygen quantum yields (Φ_Δ_) increased for cyanines but decreased for **MB** derivatives. Dye uptake experiments highlighted the importance of membrane permeability, especially given the substantially reduced accumulation and phototoxicity of the highly hydrophilic cyanine **ICy7SO_3_H**.

Future studies will focus on evaluating the phototoxicity of these dyes against **AmpB**-resistant fungal pathogens in vivo, alongside expanded investigations of structure-activity relationships (SAR) to optimize antifungal efficacy.

## Figures and Tables

**Table 1:** Spectral maxima (λ_max_Ab and λ_max_Fl), extinction coefficients (ε), fluorescence quantum yields (Φ_F_), relative yields of the ROS generation (φ_ROS_), and absolute yields of singlet oxygen generation (Φ_Δ_) in physiological saline ^1^.

Dye	Dye Structure	λ_max_Ab, nm	ε, M^−1^cm^−1^	λ_max_Fl, nm	Φ_F_, %	φ_ROS_ ^2^	Φ_Δ_ ^3^ in Saline, %	Φ_Δ_ ^4^ in MeOH, %
**Cy7CO_2_H**	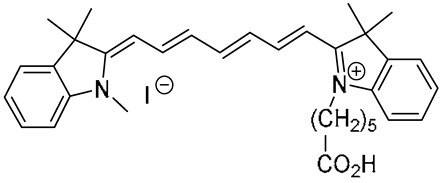	740	169,000	768	13 ± 1	0.010 ± 0.004	9.5 ± 0.6	1.1 ± 0.1
**ICy7CO_2_H**	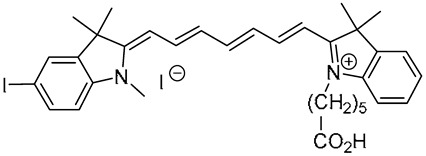	747	143,000	776	11 ± 2	1	11.8 ± 0.7	1.8 ± 0.2
**ICy7CONHPr**	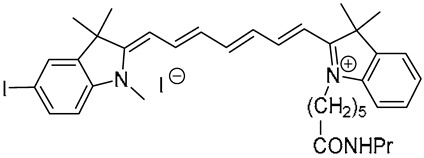	747	109,000	776	8 ± 1	0.8 ± 0.1	14.1 ± 0.5	2.5 ± 0.1
**ICy7SO_3_H**	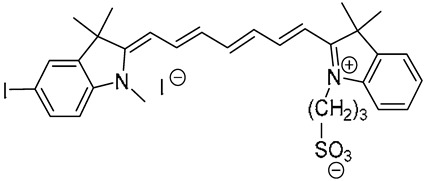	747	238,000	777	10 ± 1	2.1 ± 0.2	8.3 ± 0.4	2.0 ± 0.1
**2ICy7CO_2_H**	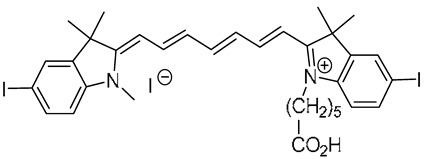	754	170,000	783	4 ± 1	2.5 ± 0.2	18.9 ± 0.6	2.3 ± 0.2

^1^ λ_max_Ab, λ_max_Fl, ε, Φ_F_, and Φ_Δ_ were collected from our previous publications [21,28], while φ_ROS_ were measured in this work. ^2^ Measured by H_2_DCFDA-based method. **ICy7CO_2_H** (φ_ROS_ = 1) was used as the reference. ^3^ Measured by SOSG-based method. ^4^ Measured in methanol by DPBF-based method.

**Table 2:** Spectral maxima (λ_max_Ab and λ_max_Fl), extinction coefficients (ε), fluorescence quantum yields (Φ_F_), relative yields of the ROS generation (φ_ROS_) measured in saline, and absolute yields of singlet oxygen generation (Φ_Δ_) in saline and acetonitrile.

Dye	Dye Structure	λ_max_Ab, nm	ε ^1^, M^−1^cm^−1^	λ_max_Fl, nm	Φ_F_, %	φ_ROS_ ^2^	Φ_Δ_ ^3^ in Saline, %	Φ_Δ_ ^4^ in AcCN, %
**MB**	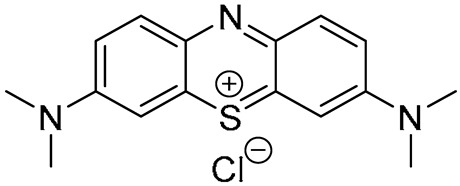	665	74,000 (88,200)	686	2.0 ± 0.5 [39]	1	60.5 ± 0.8	52 [40]
**IMB**	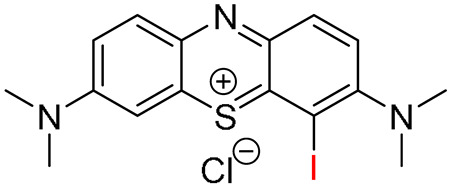	669	30,000 (35,300)	690	0.021 ± 0.004	2.4 ± 0.6	5.6 ± 0.4	25 ± 3
**2IMB**	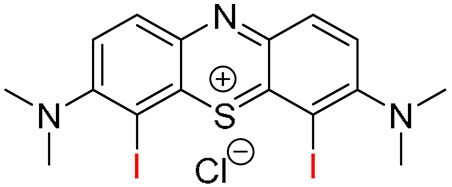	685	24,000 (30,000)	730	0.002 ± 0.001	5.5 ± 0.9	0.6 ± 0.1	20 ± 2

^1^ Extinction coefficients in MeOH are in brackets. ^2^ Measured by H_2_DCFDA-based method. **MB** (φ_ROS_ = 1) was used as the reference. The φ_ROS_ for **MB** is insufficiently higher (by only ~0.01) compared with the Φ_Δ_ [40]. ^3^ Measured in saline by SOSG-based method. ^4^ Measured in acetonitrile by DPBF-based method. **MB** (Φ_Δ_ = 52%) was taken as the reference.

**Table 3:** Uptake of dyes (*c*_Dye_ = 0.1 µM) by *C*. *albicans* and *C*. *parapsilosis* after 30 min of incubation.

Photosensitizer	Dye Uptake by *C*. *albicans* (%)	Dye Uptake by *C*. *parapsilosis* (%)
**Cy7CO_2_H**	84 ± 2	81 ± 2
**ICy7CO_2_H**	75 ± 1	70 ± 3
**ICy7CONHPr**	80 ± 2	79 ± 2
**ICy7SO_3_H**	28 ± 1	26 ± 1
**2ICy7CO_2_H**	87 ± 3	81 ± 4
**MB**	69 ± 2	68 ± 3
**IMB**	91 ± 2	94 ± 2
**2IMB**	93 ± 4	88 ± 2

**Table 4:** LC_50_ values for **AmpB** and PDT treatment of *C*. *albicans* and *C*. *parapsilosis*.

Compound	LC_50_ [µM] *C*. *albicans*	LC_50_ [µM] *C*. *parapsilosis*
**AmpB**	0.23 ± 0.09	NT ^1^
**ICy7CONHPr**	0.06 ± 0.01	0.12 ± 0.01
**2ICy7CO_2_H**	0.020 ± 0.003	0.21 ± 0.02
**ICy7CO_2_H**	0.30 ± 0.01	0.45 ± 0.09
**Cy7CO_2_H**	NT ^1^	NT ^1^
**ICy7SO_3_H**	NT ^1^	NT ^1^
**MB**	NT ^1^	NT ^1^
**IMB**	0.45 ± 0.09	0.34± 0.03
**2IMB**	0.18 ± 0.04	0.23 ± 0.03

^1^ NT—Non-toxic. LC_50_ value was not determined because of statistically insufficient cytotoxicity.

## Data Availability

The original contributions presented in this study are included in the article/Supplementary Materials. Further inquiries can be directed to the corresponding authors.

## Supplementary Materials

The following supporting information can be downloaded at https://www.mdpi.com/article/10.3390/molecules30234652/s1. **Figure S1.** Time-dependent absorption spectra of the singlet oxygen scavenger DPBF in the presence of **MB** (***A***), **IMB**, (***B***), and **2IMB** (***C***) in acetonitrile, and without PS as a control (***D***); **Figure S2.** Time-dependent fluorescence spectra of the singlet oxygen probe SOSG in the presence of **Cy7CO_2_H** (***A***), **ICy7CO_2_H** (***B***), **2ICy7CO_2_H** (***C***), **ICy7SO_3_H** (***D***), and **ICy7CONHPr** (***E***) in saline, and (***F***) and without PS as a control under light irradiation (730 nm); **Figure S3.** Time-dependent fluorescence spectra of the singlet oxygen probe SOSG in the presence of **MB** (**A**), **IMB** (**B**), and **2IMB** (**C**) in saline under light irradiation (632 nm); **Figure S4.** Time-dependent fluorescence spectra of the ROS probe H_2_DCFDA in the presence of **Cy7CO_2_H** (***A***), **ICy7CO_2_H** (***B***), **2ICy7CO_2_H** (***C***) **ICy7SO_3_H**, (***D***), and **ICy7CONHPr** (***E***) in saline, and without PS as a control (***F***) under light irradiation (730 nm); **Figure S5.** Time-dependent fluorescence spectra of the ROS probe H_2_DCFDA in the presence of **MB** (**A**), **IMB** (**B**), and **2IMB** (**C**) in saline, and without PS as a control (**D**) under light irradiation (632 nm); **Figure S6**. ^1^H NMR spectrum (400 MHz, CDCl_3_) of compound **2IMB** at 25 °C; **Figure S7**. Theoretical (top) and experimental (bottom) HRMS spectrum of **2IMB**; **Figure S8**. HPLC chromatogram of **2IMB**; **Figure S9**. 1H NMR spectrum (400 MHz, DMSO-d6) of compound **IMB** at 25 °C; **Figure S10**. Theoretical (top) and experimental (bottom) HRMS spectrum of **IMB**; **Figure S11**. HPLC chromatogram of **IMB**; **Figure S12**. MS spectrum of **IMB**; **Figure S13**. MS spectrum of **2IMB**.
