# Supplementary material for: Iodinated Near-Infrared Dyes as Effective Photosensitizers for the Photodynamic Eradication of Amphotericin B-Resistant Candida Pathogens
Source: Molecules. 2025 Dec 4;30(23):4652. doi: 10.3390/molecules30234652 (PMC12692796; doi:10.3390/molecules30234652)
Supplement: Supplementary file 1 [file molecules-30-04652-s001.zip › molecules-4002413-supplementary.pdf]

Iodinated Near-Infrared Dyes as Effective Photosensitizers for the Photodynamic Eradication of Amphotericin B-Resistant Candida Pathogens

Chen Damti [1], Andrii Bazylevich [1], Amartya Sanyal [1], Olga Semenova [1], Arjun Prakash [1], Iryna Hovor [1,2], Bat Chen R. Lubin [2,3], Leonid Patsenker [4] and Gary Gellerman [1,*]

[1] Department of Chemical Sciences, Faculty of Natural Sciences, Ariel University, Ariel, 40700, Israel; chendrori27@gmail.com (C.D.); andriib@ariel.ac.il (A.B.); amartya@ariel.ac.il (A.S.); olgasem@ariel.ac.il (O.S.); arjunvprakash22@gmail.com (A.P.); irynah@ariel.ac.il (I.H.)
[2] Department of Chemical Engineering, Biotechnology and Materials, Ariel University, Ariel 40700, Israel; revital5@gmail.com
[3] Agriculture and Ecology Department, Eastern Regional R&D Center, Ariel 40700, Israel
[4] Independent researcher, Ariel, 40700, Israel; leonid.patsenker@gmail.com
* Correspondence: garyg@ariel.ac.il; Tel.: 972-74-7233079

Spectral data

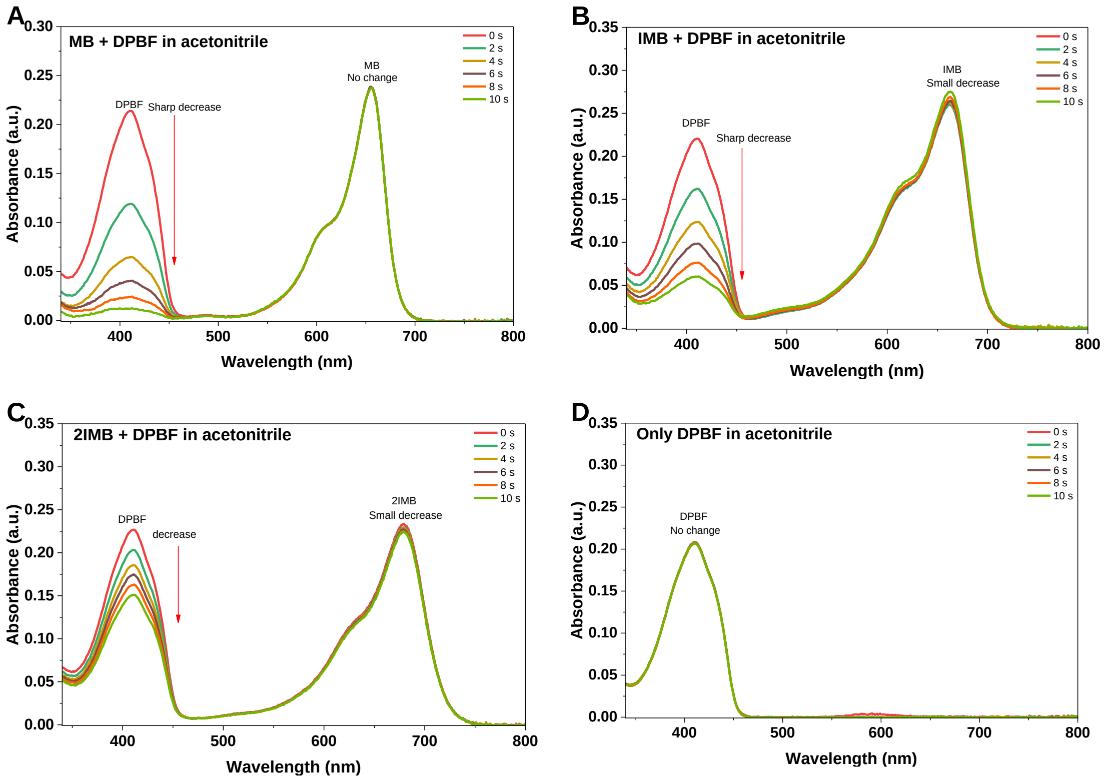

Figure S1. Time-dependent absorption spectra of the singlet oxygen scavenger DPBF in the presence of **MB** (***A***), **IMB**, (***B***), and **2IMB** (***C***) in acetonitrile, and without PS as a control (***D***). The progressive decrease of the DPBF band (at 410 nm) under light irradiation (632 nm) indicates photooxidation resulting from PS-induced $^1O_2$ generation. The control shows no spectral changes over the irradiation time.

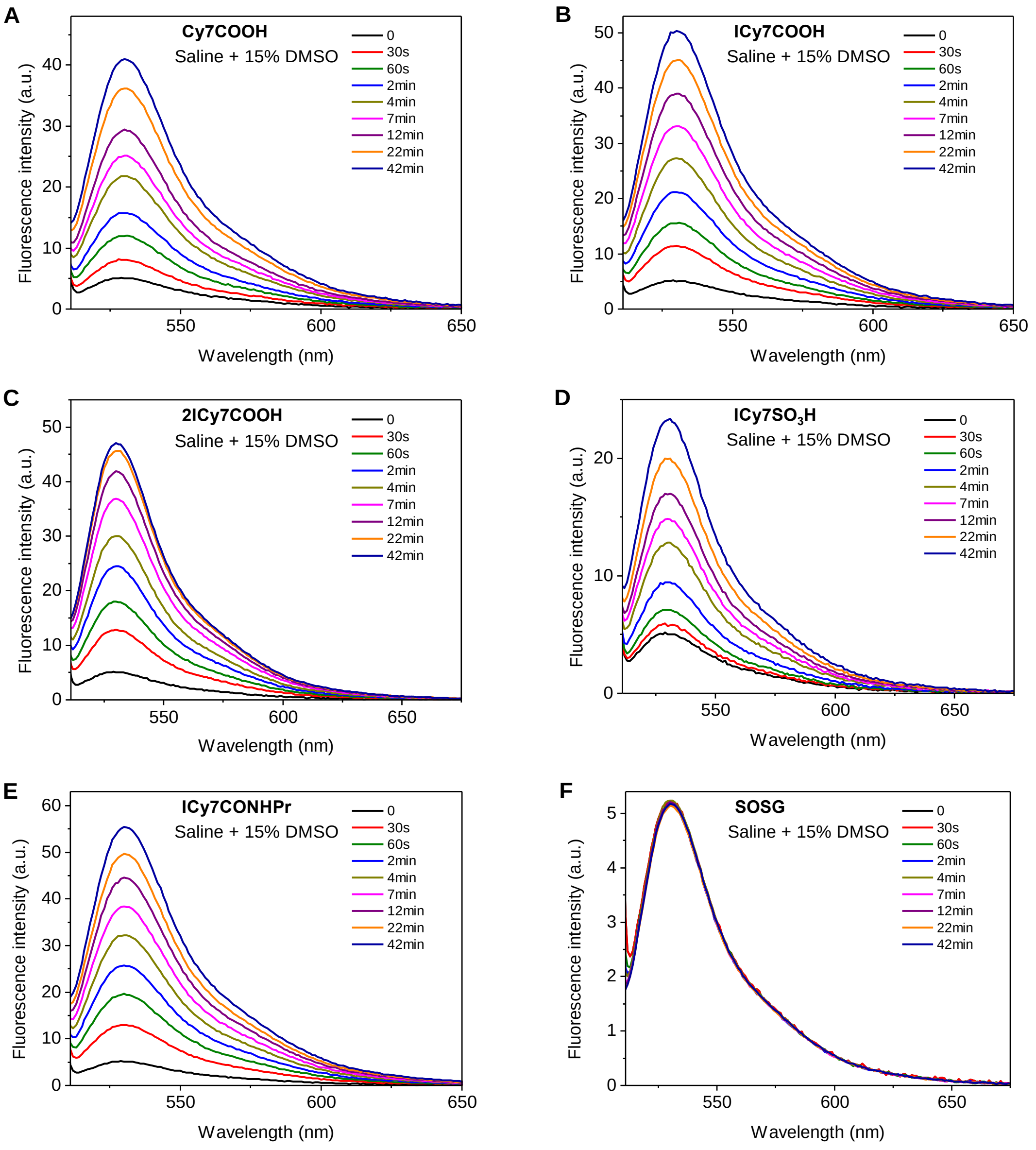

Figure S2. Time-dependent fluorescence spectra of the singlet oxygen probe SOSG in the presence of **Cy7CO₂H** (***A***), **ICy7CO₂H** (***B***), **2ICy7CO₂H** (***C***), **ICy7SO₃H** (***D***), and **ICy7CONHPr** (***E***) in saline, and (***F***) and without PS as a control under light irradiation (730 nm). The progressive increase in SOSG fluorescence (at 530 nm) indicates singlet oxygen generation. The control solution of SOSG shows no detectable changes.

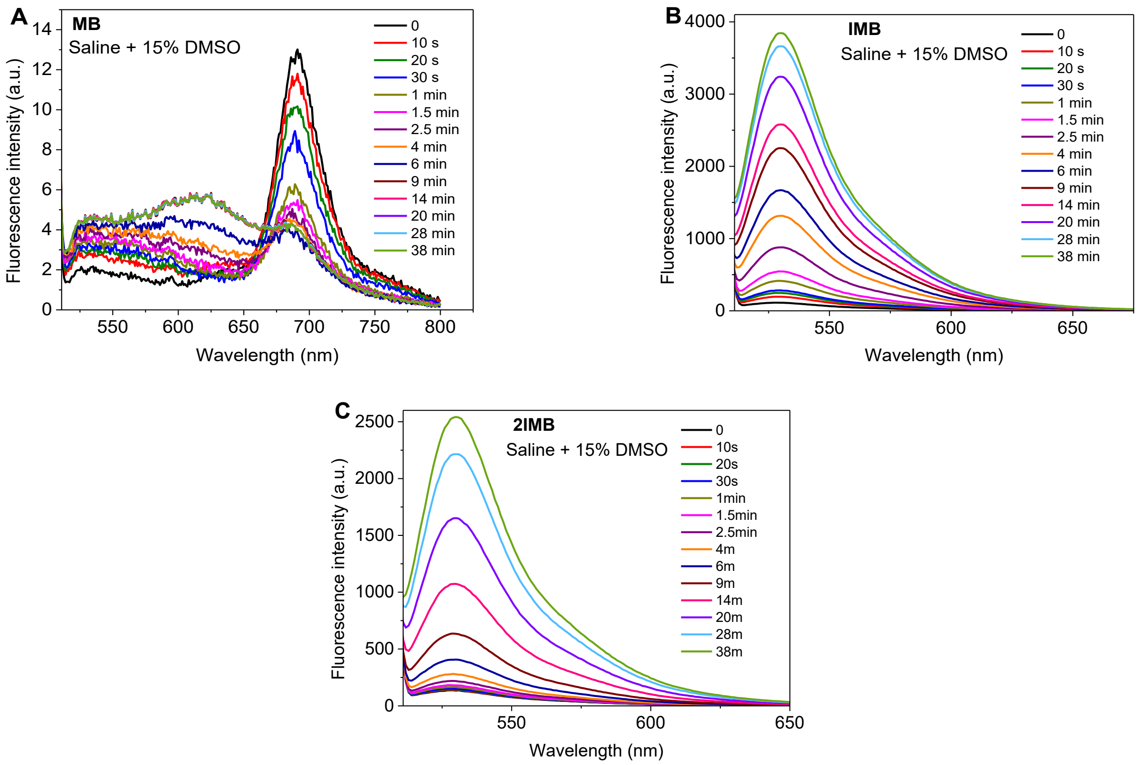

Figure S3. Time-dependent fluorescence spectra of the singlet oxygen probe SOSG in the presence of **MB** (**A**), **IMB** (**B**), and **2IMB** (**C**) in saline under light irradiation (632 nm). The progressive increase in SOSG fluorescence (at 530 nm) indicates singlet oxygen generation.

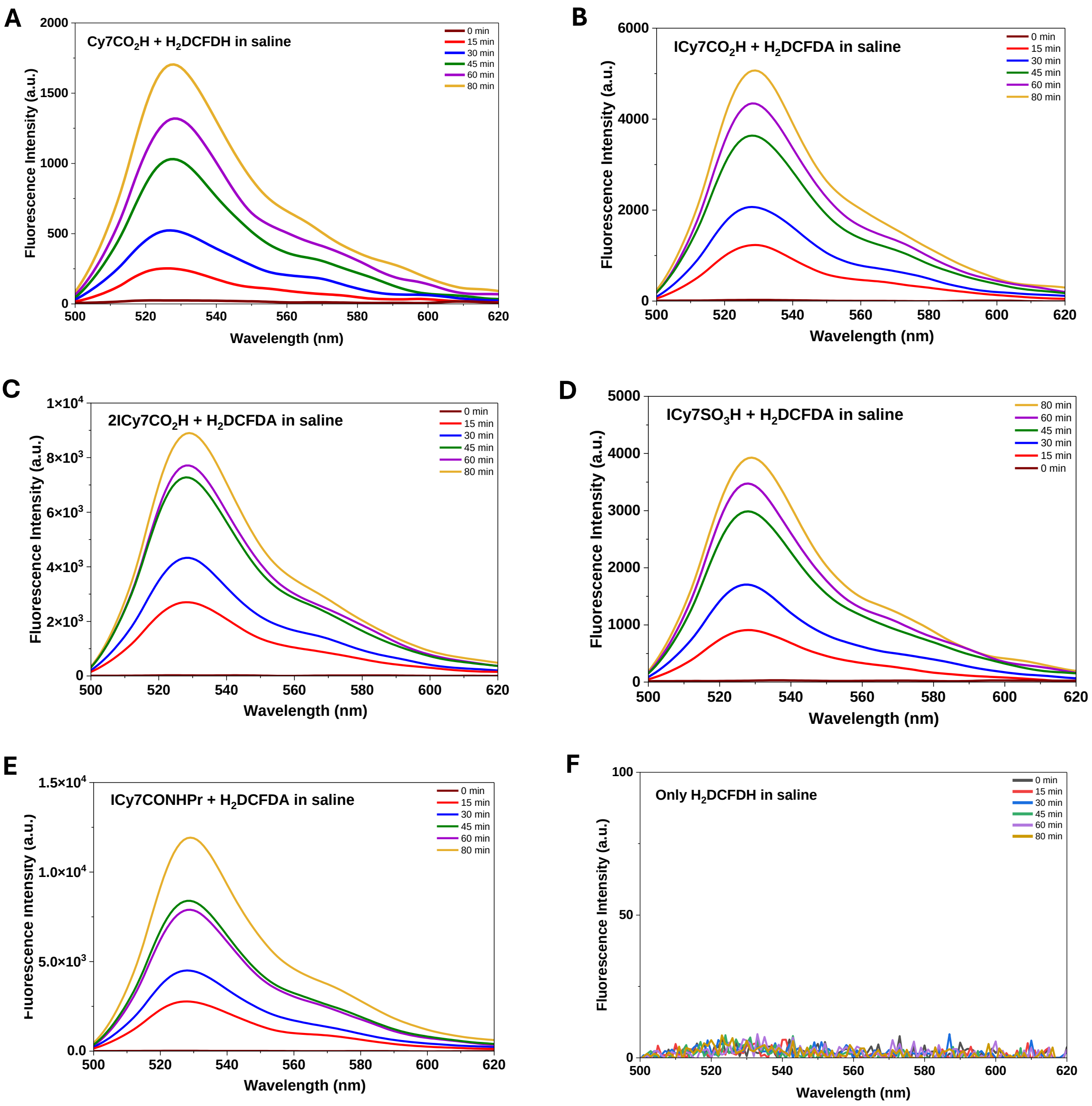

Figure S4. Time-dependent fluorescence spectra of the ROS probe H_2DCFDA in the presence of **Cy7CO$_2$H** (***A***), **ICy7CO$_2$H** (***B***), **2ICy7CO$_2$H** (***C***) **ICy7SO$_3$H**, (***D***), and **ICy7CONHPr** (***E***) in saline, and without PS as a control (***F***) under light irradiation (730 nm). The progressive increase in DCF fluorescence (at 525 nm) indicates ROS generation. The control shows almost no detectable changes.

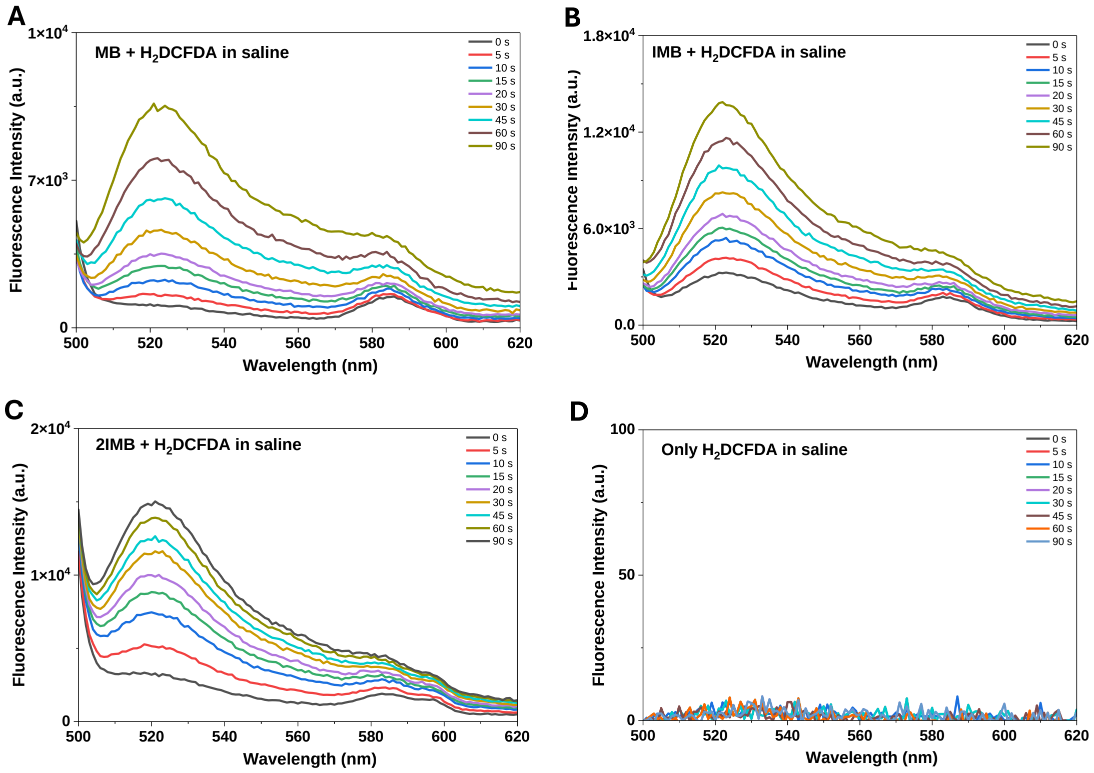

Figure S5. Time-dependent fluorescence spectra of the ROS probe H_2DCFDA in the presence of **MB** (***A***), **IMB** (***B***), and **2IMB** (***C***) in saline, and without PS as a control (**D**) under light irradiation (632 nm). The increasing DCF fluorescence (at 525 nm) indicates ROS generation. The control shows almost no detectable changes.

^{1}H NMR of 2IMB

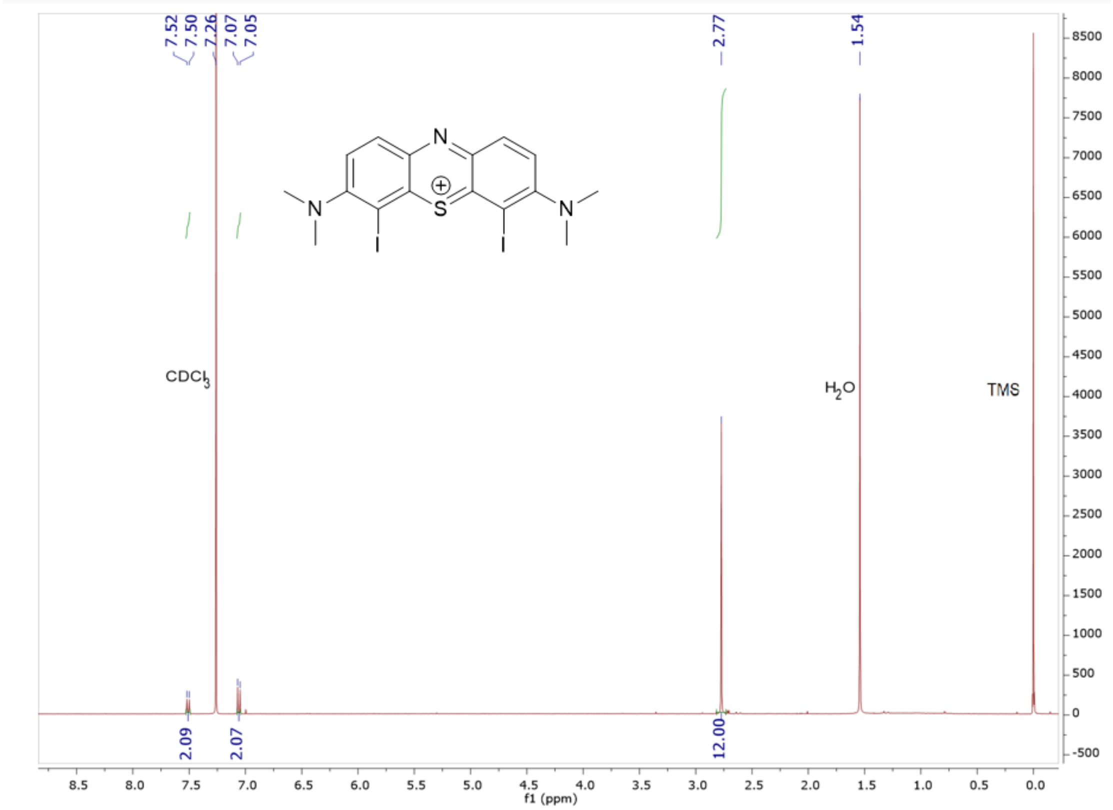

Figure S6. ^{1}H NMR spectrum (400 MHz, $CDCl_3$) of compound **2IMB** at 25 °C.

HRMS spectra of 2IMB

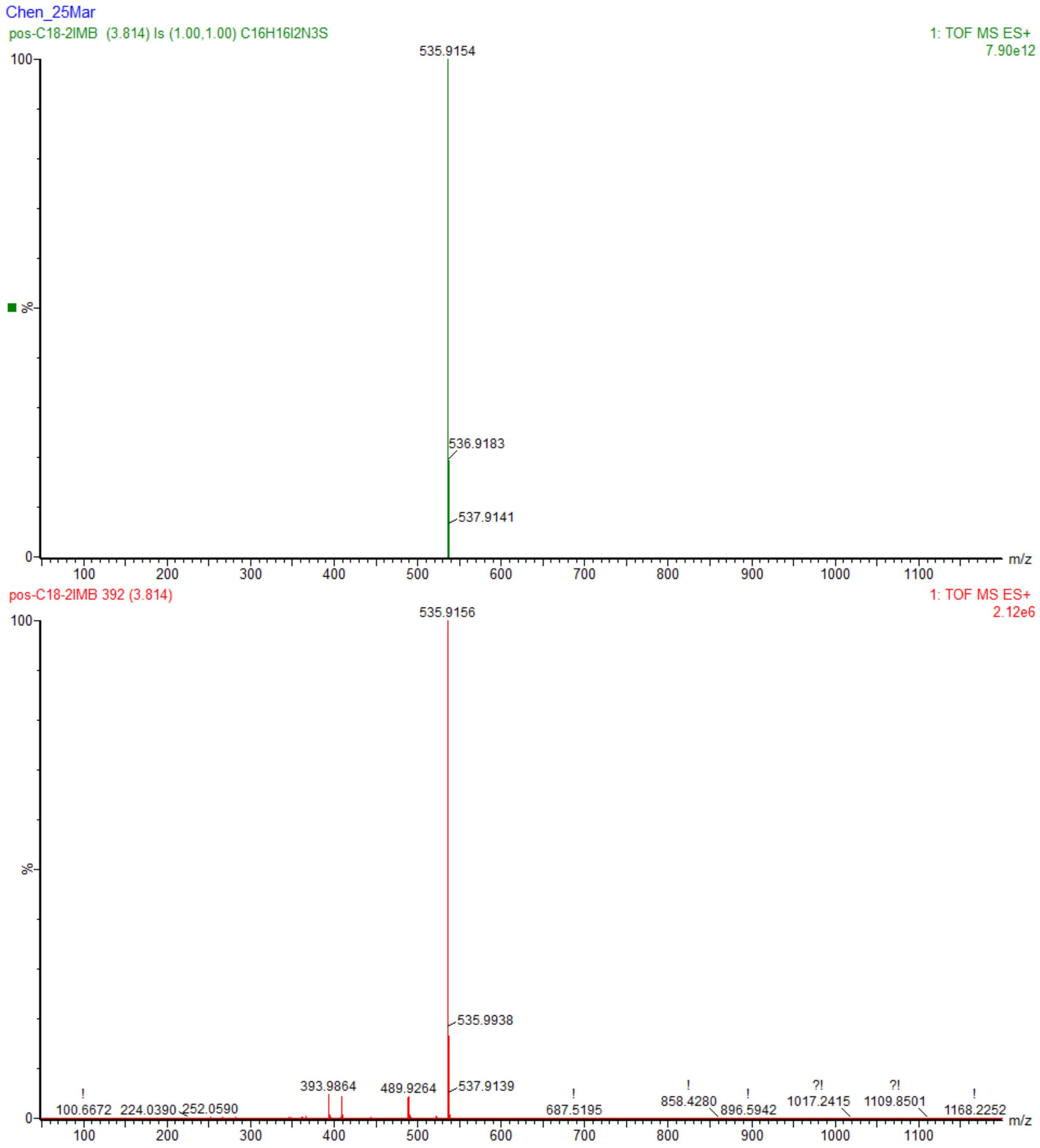

Figure S7. Theoretical (top) and experimental (bottom) HRMS spectrum of **2IMB**.

HPLC of 2IMB

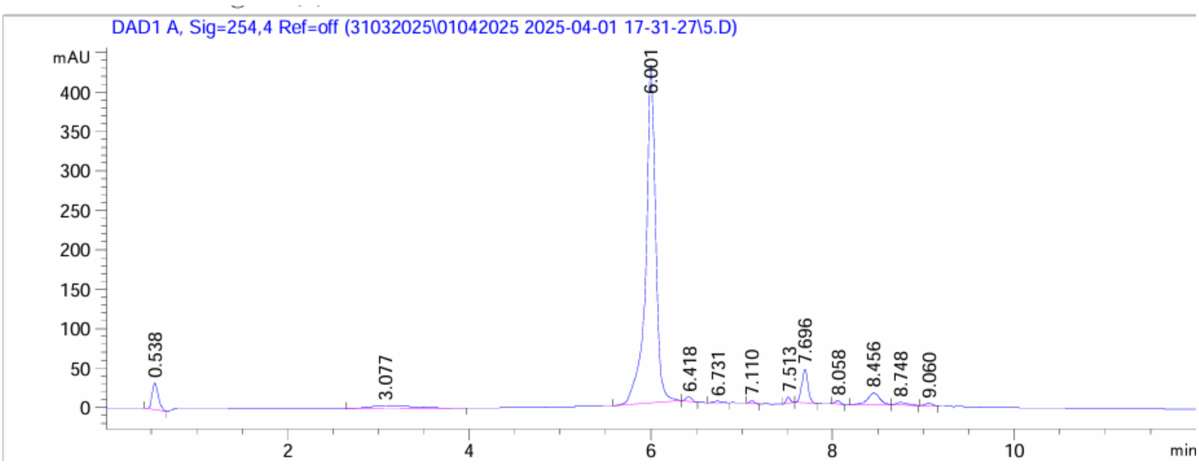

Figure S8. HPLC chromatogram of **2IMB**.

^{1}H NMR spectrum of IMB

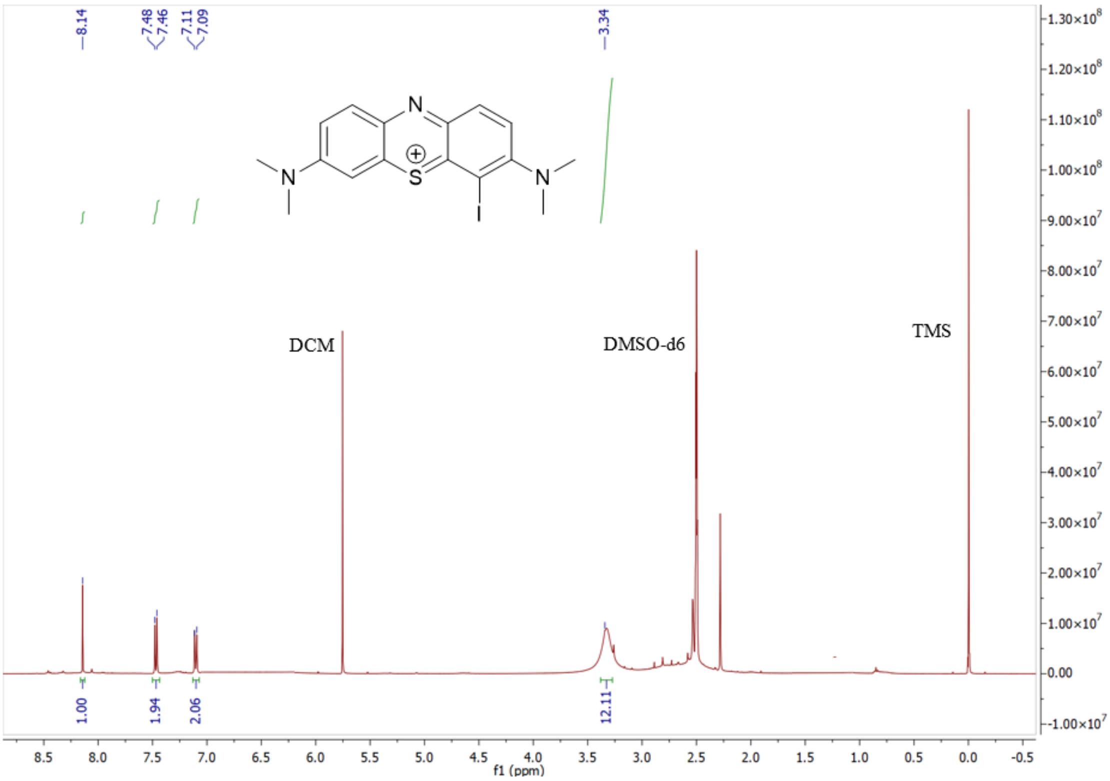

Figure S9. ^{1}H NMR spectrum (400 MHz, DMSO-d6) of compound **IMB** at 25 °C.

HRMS spectra of IMB

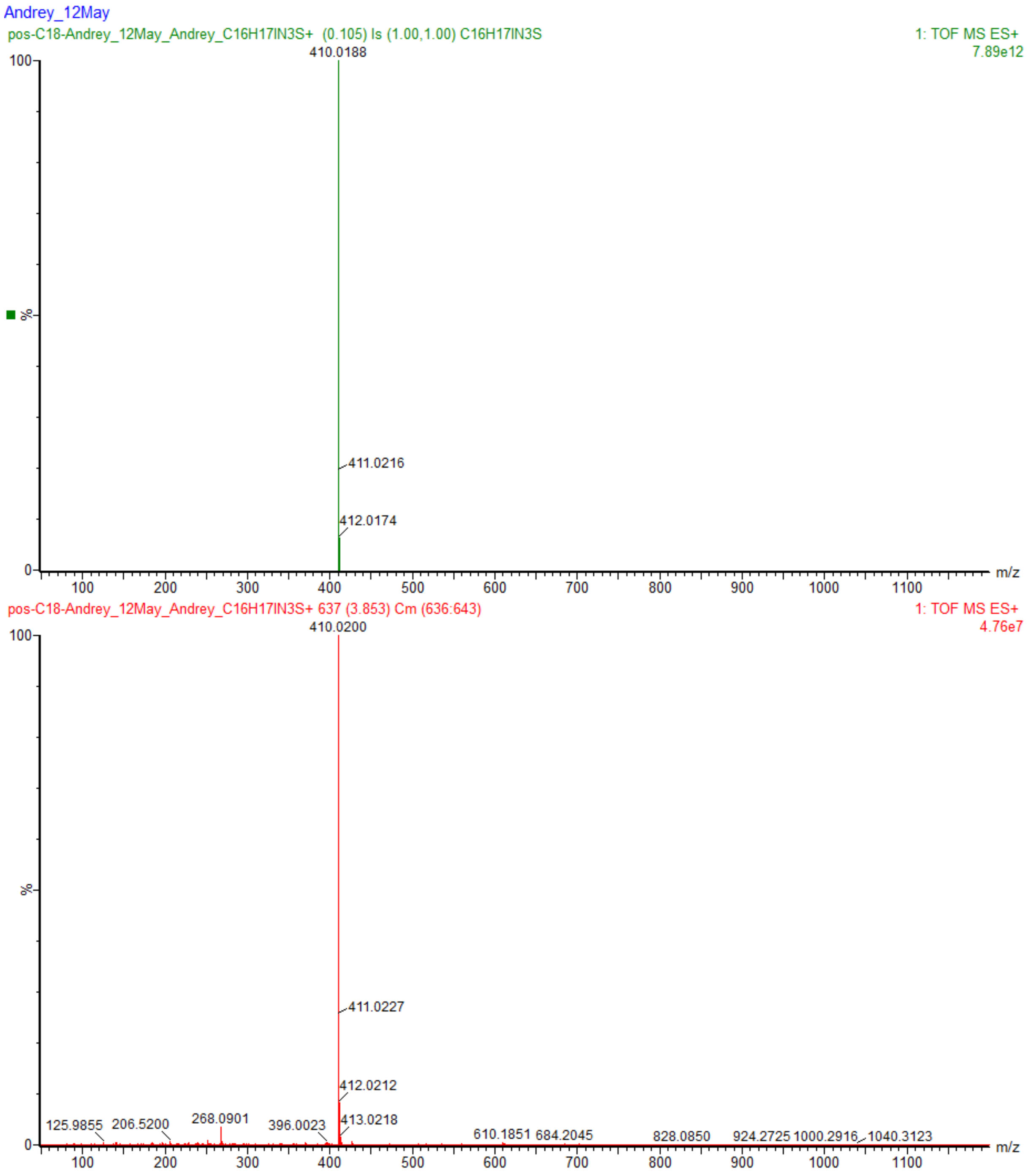

Figure S10. Theoretical (top) and experimental (bottom) HRMS spectrum of **IMB**.

HPLC of IMB

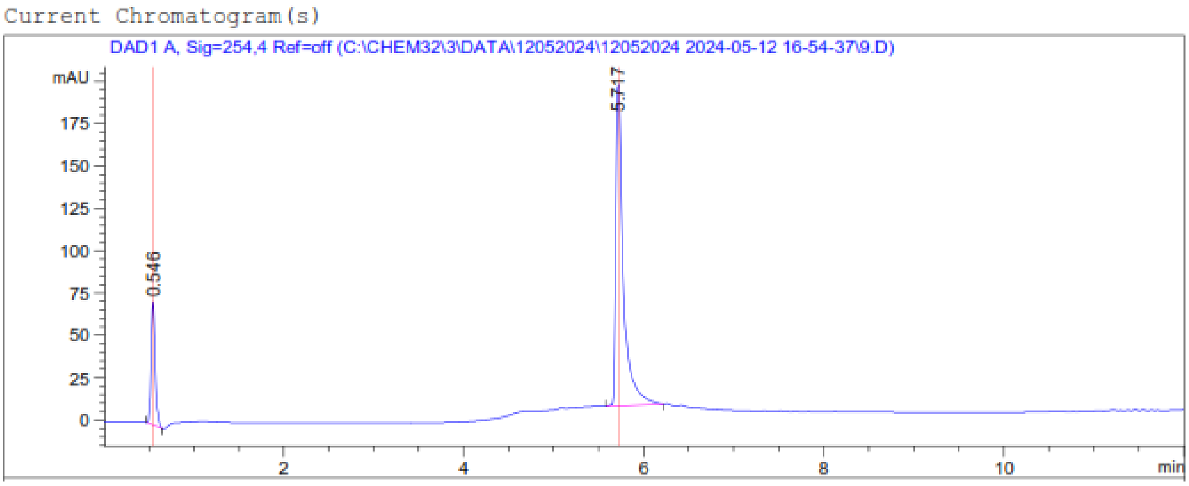

Figure S11. HPLC chromatogram of **IMB**.

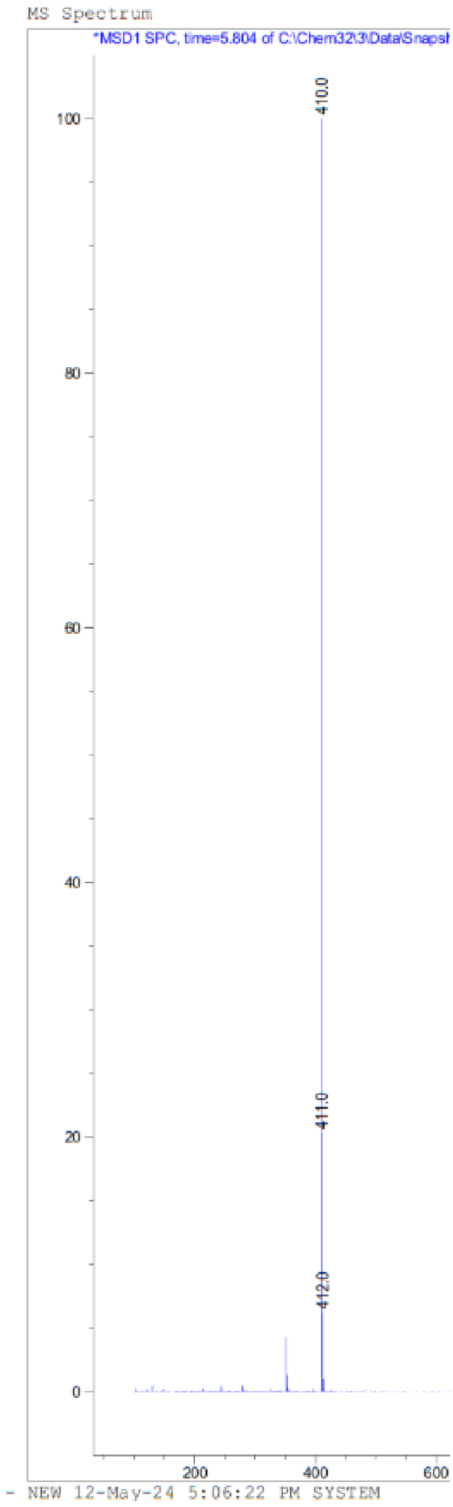

Figure S12. MS spectrum of **IMB**.

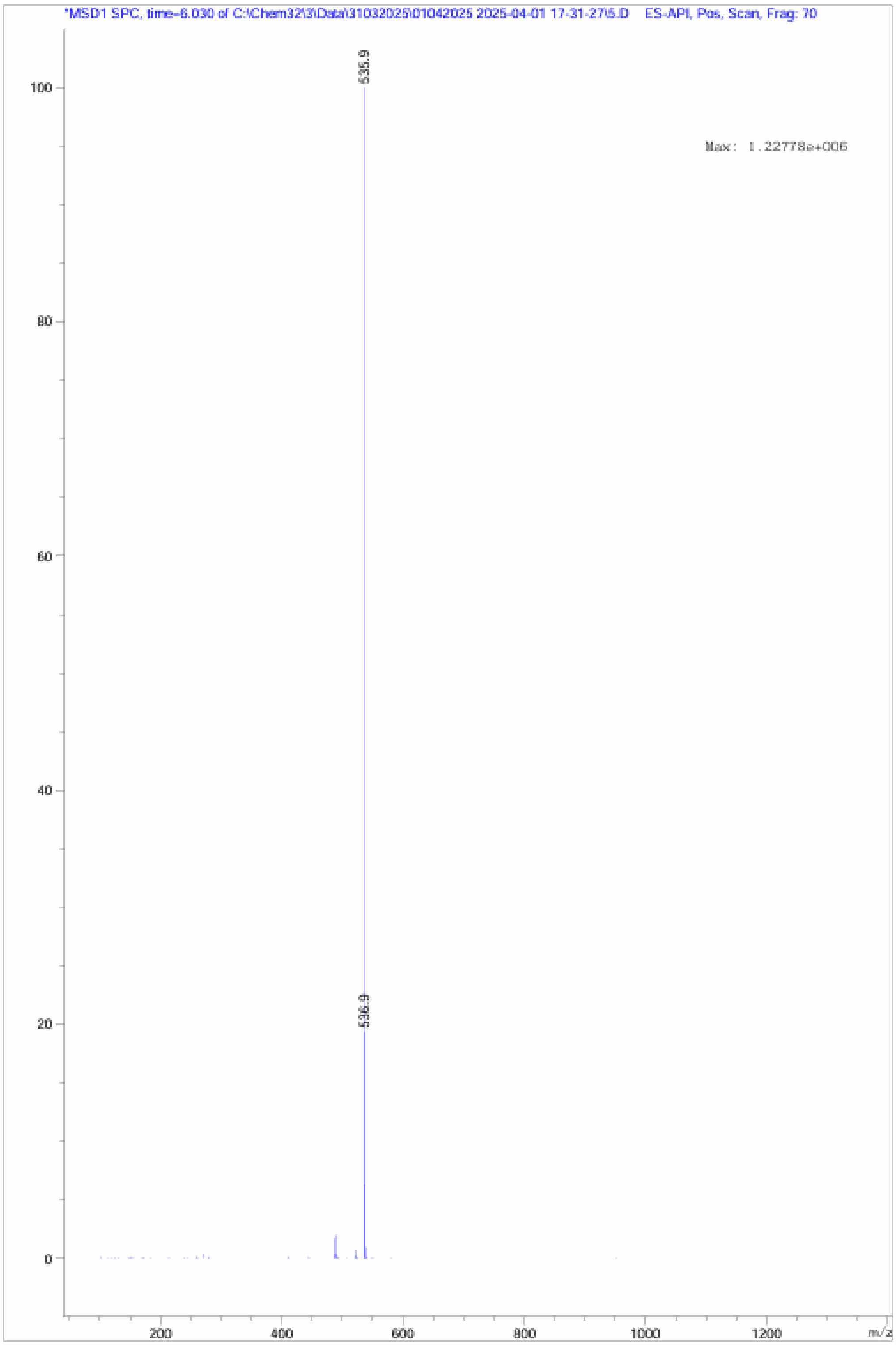

Figure S13. MS spectrum of **2IMB**.
